# Identification and validation of an epigenetically regulated long noncoding RNA model for breast cancer metabolism and prognosis

**DOI:** 10.1186/s12920-022-01256-2

**Published:** 2022-05-07

**Authors:** Yu Song, Songjie Shen, Qiang Sun

**Affiliations:** Department of Breast Surgery, Peking Union Medical College Hospital, Chinese Academy of Medical Sciences and Peking Union Medical College, No.1 Shuaifuyuan Street, Dongcheng District, Beijing, China

**Keywords:** Breast cancer, lncRNA signature, Methylation, Prognosis, Metabolism

## Abstract

**Background:**

Breast cancer (BC) is the leading cause of death among women, and epigenetic alterations that can dysregulate long noncoding RNAs (lncRNAs) are thought to be associated with cancer metabolism, development, and progression. This study investigated the epigenetic regulation of lncRNAs and its relationship with clinical outcomes and treatment responses in BC in order to identify novel and effective targets for BC treatment.

**Methods:**

We comprehensively analysed DNA methylation and transcriptome data for BC and identified epigenetically regulated lncRNAs as potential prognostic biomarkers using machine learning and multivariate Cox regression analysis. Additionally, we applied multivariate Cox regression analysis adjusted for clinical characteristics and treatment responses to identify a set of survival-predictive lncRNAs, which were subsequently used for functional analysis of protein-encoding genes to identify downstream biological pathways.

**Results:**

We identified a set of 1350 potential epigenetically regulated lncRNAs and generated a methylated lncRNA dataset for BC, MylnBrna, comprising 14 lncRNAs from a list of 20 epigenetically regulated lncRNAs significantly associated with tumour survival. MylnBrna stratifies patients into high-risk and low-risk groups with significantly different survival rates. These lncRNAs were found to be closely related to the biological pathways of amino acid metabolism and tumour metabolism, revealing a potential tumour-regulation function.

**Conclusion:**

This study established a potential prognostic biomarker model (MylnBrna) for BC survival and offered an insight into the epigenetic regulatory mechanisms of lncRNAs in BC in the context of tumour metabolism.

**Supplementary Information:**

The online version contains supplementary material available at 10.1186/s12920-022-01256-2.

## Introduction

Breast cancer (BC) is the most common malignancy in women and the leading cause of cancer-related deaths worldwide. BC, lung cancer, and colorectal cancer account for 50% of all new cancer diagnoses among women, whereas BC alone accounts for 30% of female cancers [1]. Despite the rigorous selection of multiple treatment options to prolong patient survival according to each individual patient, many patients continue to experience BC recurrence and metastasis due to treatment resistance and wide variations in individual genetic specificity [2]. BC can be difficult to treat owing to its genetic and molecular heterogeneity, especially in metabolically active or recurrent cases. Growing evidence suggests that altered molecular profiles offer insights into possible therapeutic approaches to improve cancer diagnosis, prognosis, and response to therapy [3]. Recently, long noncoding RNAs (lncRNAs) have become a hotspot in the field of biomarker research and have been extensively studied and characterized in various cancers. Aberrant expression of lncRNAs has also been observed in BC development, progression, recurrence, metastasis, treatment resistance, and targeted therapy [4]. Several lncRNA expression signatures have been proposed for predicting and monitoring disease status, prognosis, and drug sensitivity [5]. However, epigenetic regulation of lncRNAs and their potential function and clinical applications in BC, particularly their specific involvement in tumour metabolism, require further investigation. Therefore, a deeper understanding of the molecular mechanisms associated with BC tumorigenesis is critical.

Metabolic reprogramming is a primary characteristic of many cancer types [6], and BC cells exhibit distinct metabolic plasticity to fuel their proliferation and progression [7–9]. Reprogramming of cancer cell metabolism is considered a ground-breaking hallmark that actively contributes to cancer development [10–12]. Through epigenetic regulation, metabolism actively contributes to tumorigenesis via integrated metabolite production, interactions with signalling pathways, and metabolite dependence. Alterations in the metabolic program of cancer cells further affect other cells in the tumour microenvironment and are involved in regulating other processes closely associated with cancer development, such as angiogenesis, inflammation, and cancer immunity [11, 13]. Oncogenic events drive dysregulation of metabolic pathways to provide a selective advantage for cancer cells to proliferate and survive in the hostile microenvironment. Thus, analysis of the metabolic regulation and altered characteristics of BC cells may reveal key vulnerabilities in the disease and identify new diagnostic and therapeutic perspectives.

LncRNAs constitute a class of noncoding RNAs with lengths > 200 nucleotides and minimal evidence of protein-coding ability and are crucial players in a variety of cellular and physiological functions [14]. Accumulating evidence has revealed that dysregulated expression of lncRNAs is involved in tumour initiation, progression, and metastasis [15]. In BC, lncRNAs are emerging as master regulators of tumour biology, with oncogenic functions associated with tumorigenesis and tumour progression (HOTAIR, MALAT-1, lincRNAp21, and GAS5) [16]. A recent study identified the hypoxia-responsive lncRNA BCRT1 as a tumour promoter in BC, with its expression unfavourably associated with tumour metastasis and poor prognosis according to its involvement in sponging microRNAs through exosome-mediated transfer [17]. Another study showed that lncRNA DILA1 overexpression increases tamoxifen resistance in BC by inhibiting the degradation of cyclin D1 [4]. Dysregulation of these lncRNAs is associated with biological functions such as invasion, proliferation, apoptosis, and cell cycle progression as well as clinical features such as cell survival, tumour progression, and risk of metastasis. Mechanisms related to oncogenic and tumour-suppressive pathways are modulated by lncRNAs with direct or indirect effects, including gene expression regulation, chromatin remodelling, post-transcriptional regulation, and translational control [18, 19]. Mapping the expression patterns and action mechanisms of lncRNAs is of great value and may contribute to identification of new biomarkers for BC diagnosis as well as targets of potential therapies.

Gene methylation and epigenetics in tumour cells, especially aberrant gene methylation, has been detected in a variety of cancer types, involving coding and noncoding genes for a variety of crucial tumour functions such as the cell cycle, DNA repair, toxic compound catabolism, cell adhesion, apoptosis, and angiogenesis [20, 21]. Recently a study reported that a DNA methylation model based on 11 DNA methylation biomarkers was developed and validated for use in clinical practice to detect early colorectal cancer [22]. Currently, N^6^-Methyladenosine (m^6^A) is the most common and widely researched mRNA modification that affects diverse biological processes in a reversible manner and involves regulation of protein expression through “writers,” “erasers,” and “readers” [23]. Because RNA m^6^A modifications are involved in gene expression regulation and various biological processes, it is reasonable to believe that aberrant RNA modifications play an important role in carcinogenesis. Increasing evidence suggests that noncoding RNAs also actively affect signalling networks within tumour cells [22, 24, 25]; therefore, it is reasonable to suggest that lncRNA methylation, which is closely related to lncRNA expression and function, plays a key role in oncogenesis. While data on DNA methylation in BC has been reported, lncRNA methylation in BC has not been extensively studied. Studies on lncRNA modifications have been the focus of many investigations into BC progression and drug resistance. A recent study found that aberrant activation of the histone methyltransferase EZH2 promotes ribosome synthesis by regulating and silencing lncRNA PHACTR2-AS1, which leads to over-activation of ribosome synthesis and instability of ribosomal DNA, promoting BC metastasis [26]. However, the mechanism by which “writers” or “erasers” regulate lncRNA methylation requires further investigation.

In this study, we explored the landscape of lncRNA transcription mediated by differential methylation in BC and investigated its association with tumour metabolism. Furthermore, analyses of the prognostic effects of these lncRNAs on BC-specific therapeutic responses identified potential prognostic biomarkers that will offer insights for subsequent tumour studies and possible clinical applications to improve patient survival.

## Methods

### Acquisition and analysis of DNA methylation data for patients with BC

Methylation data were obtained from UCSC Xena (https://xena.ucsc.edu/), pre-processed, and subsequently analysed using the R package RnBeads (https://www.bioconductor.org/packages/release/bioc/html/RnBeads.html). We then performed pre-processing and subsequent differential methylation analyses of the data. Imputation was performed by calculating the median methylation level for each sample across all CpG sites and replacing all missing values for a given sample at an individual CpG site with the median across all CpGs in the sample. Imputation replaced the median of two missing values per sample by estimations.

P-values at the site level were computed using the limma method [27]. Hierarchical linear models from the limma package (https://bioconductor.org/packages/release/bioc/html/limma.html) were employed and fitted using an empirical Bayes approach on the derived M-values. The differences in mean methylation levels between the cancer and normal tissue groups were compared, and a statistical test (limma or *t* test depending on the settings) was performed to determine whether the methylation values in the two groups originated from distinct distributions. The sites were ranked according to each metric and assigned a combined rank (each site was assigned a rank based on each of three criteria: the quotient in mean methylation levels, the quotient in mean methylation ploidy levels, and the P value for methylation), with this computed as the maximum (i.e., worst) rank among the three ranks according to the criteria (the smaller the combined rank for a site, the more evidence for differential methylation at that site). Volcano plots and heat maps of differentially methylated sites were constructed, and the methylation sites in the genomic regions were annotated (four genomic regions in total), as follows: 1) methylation values at the gene level were extracted for each site for differential methylation analysis, and 2) a volcano plot and a heat map of differentially methylated genes were constructed. Functional enrichment of differentially hypermethylated genes was performed using the R package GOstats (https://bioconductor.org/packages/release/bioc/html/GOstats.html).

### Acquisition and analysis of differentially methylated LncRNAs

Differentially methylated sites were selected according to a false discovery rate (FDR)-adjusted P < 0.05 and an absolute mean methylation difference > 0.3, and the lncRNA expression profile was obtained from UCSC Xena. The Z-score of the lncRNA expression profile was normalised, and Pearson’s correlation test between differentially methylated sites and lncRNA expression was performed. LncRNAs with an absolute correlation > 0.4, correlation P < 0.01, and with differentially methylated sites were selected for further analysis.

### Identification of prognostic LncRNA markers mediated by differential methylation

We then used the differentially methylated lncRNAs to analyse patient survival by calculating the hazard ratio (HR), 95% confidence interval (CI), Z value, and P value for each lncRNA, followed by application of the survival R package (https://cran.r-project.org/web/packages/survival/index.html) after dividing the data into a training set and test set. Specifically, to identify the optimal combination, we added variables that resulted in the greatest significant improvement and removed variables that caused the most insignificant deterioration in the quality of the prediction model at each step, assessed based on the Akaike information criterion. This process was repeated until the model no longer improved at a statistically significant level.

### Evaluation of model performance in BC prognosis

Cox regression was applied to the training set, and a risk score was generated for each patient. A median risk score of 0.375 was used as the threshold to divide patients into high-risk (> threshold) and low-risk (≤ threshold) groups for further prognostic analysis. Survival curves were generated according to the groups within the training set, including their overall survival (OS), disease-free survival (DFS), disease-specific survival (DSS), and progression-free survival (PFS). The P values were not indicated in the survival curves of the training set. We then performed univariate Cox analysis to generate a Cox regression model, with HRs, 95% CIs, Z values, and P values determined for the 14 potential lncRNA prognostic markers using multivariate Cox analysis on the training set. The same method was used for survival analysis on the test set and the overall set to evaluate the model performance in BC prognosis.

### Determination of correlations between prognostic markers and metabolism

A total of 10 major categories and 86 metabolism-related pathways previously reported in tumour cells were obtained from the Kyoto Encyclopaedia of Genes and Genomes (KEGG) database [28]. Using gene set variation analysis [29], we converted the gene expression profile data into profiles of metabolic pathway activity based on relationships between the metabolic pathways and corresponding genes. Using the limma method [27], we performed a pathway activity difference analysis for the high-risk versus low-risk groups using the dataset, with metabolic pathways having a corrected P < 0.05 considered significantly different.

### Functional enrichment analysis

To explore the functions of the potential lncRNA biomarkers, we performed functional enrichment analysis using mRNAs demonstrating correlations between their expression and the level of each lncRNA. The association between lncRNA and mRNA expression was measured by calculating the Pearson’s correlation coefficient, with the top 50 mRNAs considered “related” to each lncRNA. We then performed function enrichment analysis using Gene Ontology (GO) and KEGG analyses to infer possible functional roles of the lncRNAs using the R package clusterProfiler (https://bioconductor.org/packages/release/bioc/html/clusterProfiler.html). GO terms or KEGG pathways with an adjusted P < 0.05 were considered significantly enriched.

### Statistical analysis

All statistical analyses were performed using SPSS (v25.0; IBM Corp. Armonk, NY, USA) and R Statistical Software (v3.6.3; https://www.r-project.org/). Qualitative variables were compared using the chi-square test, survival curves were prepared according to the Kaplan‒Meier method, and survival was compared using the log-rank test. Statistical significance was set at P < 0.05.

**Fig. 1:**
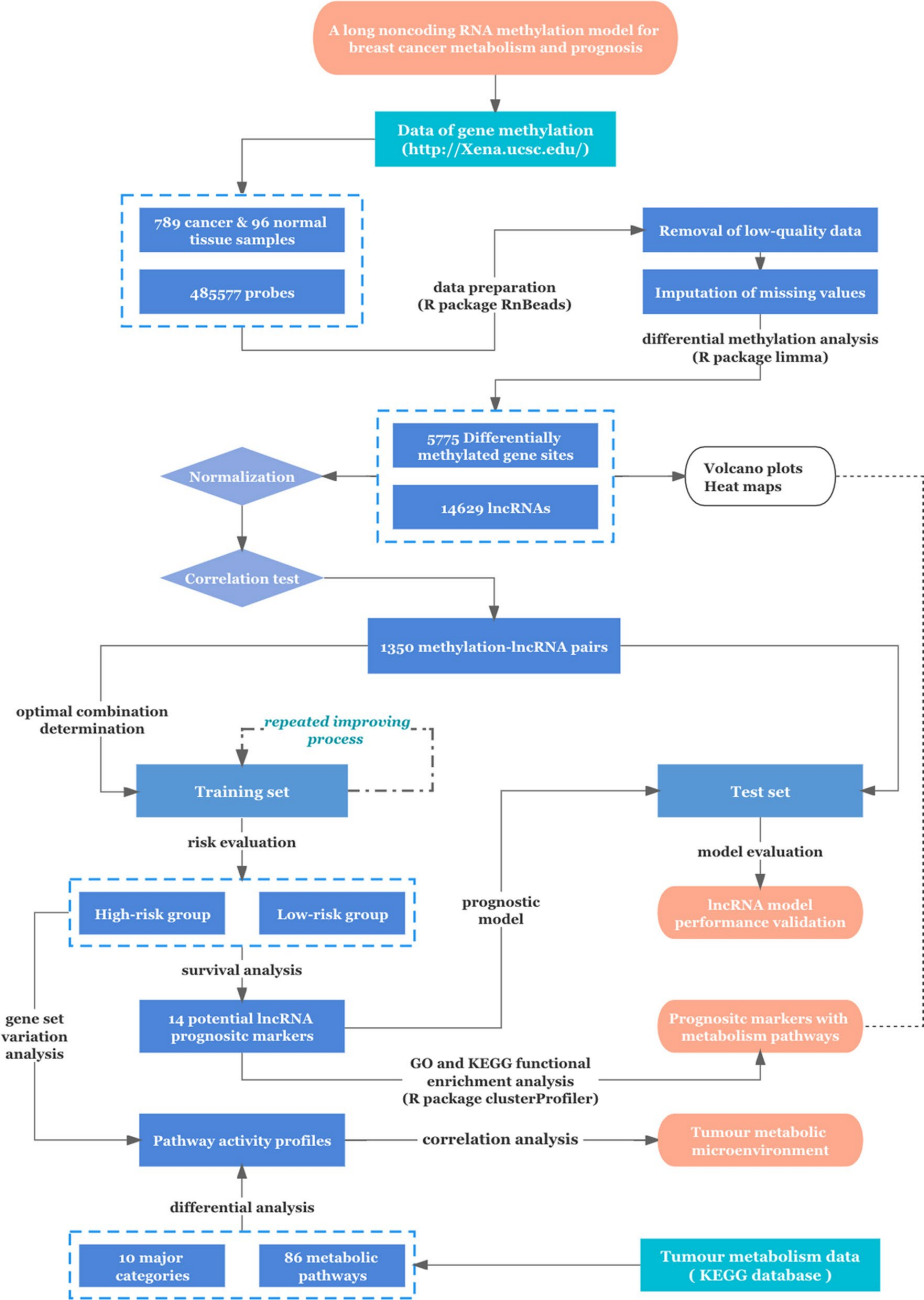
Workflow of this study

The workflow of this study is presented in Fig. 1.

## Results

### Gene expression and methylation data

**Fig. 2:**
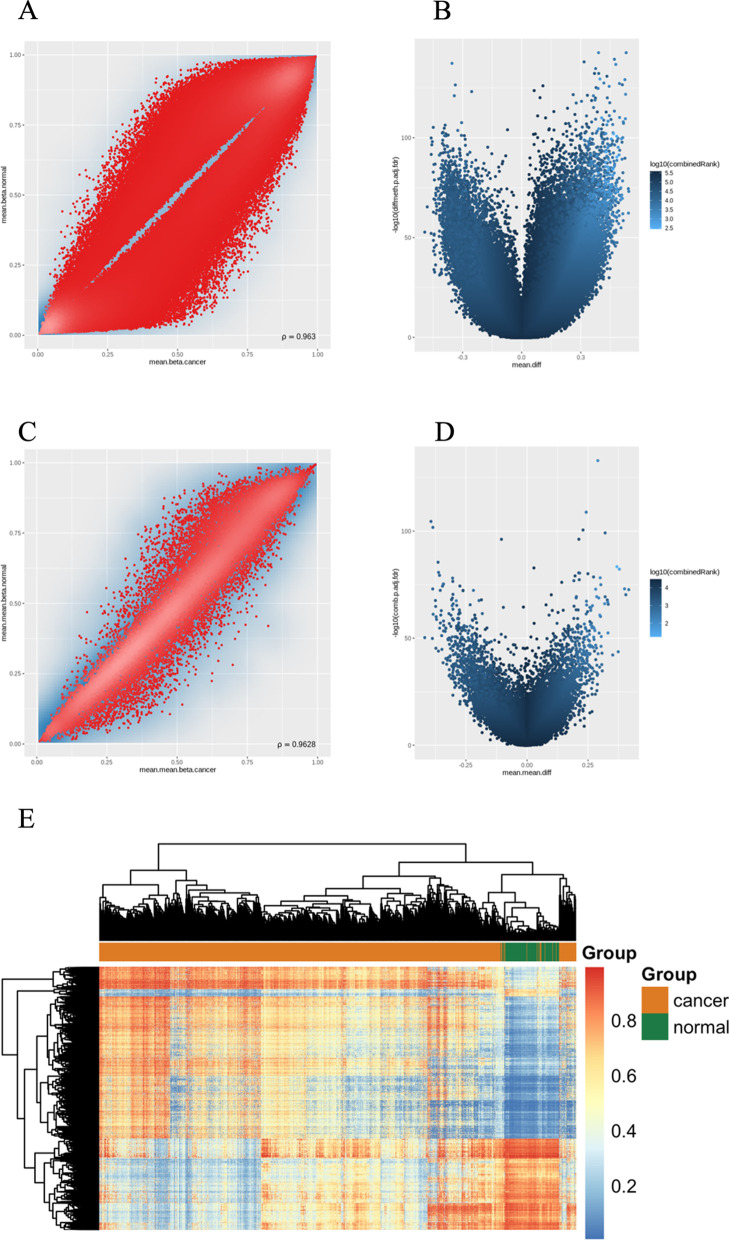
Analysis of gene expression and methylation data. **A** Distribution of methylation values for differentially methylated sites in the cancer and normal tissue groups. **B** Volcano plot of differentially methylated sites. **C** Distribution of methylation values for the differentially methylated genes in each group. **D** Volcano plot of differentially methylated genes. **E** Heat map of differentially methylated sites showing distribution of methylation values between the two groups (one row for a site, and one column for a sample)

BC methylation data were obtained from UCSC Xena, including a total of 485,577 probes and 885 samples (789 tumour samples and 96 normal samples). Of these, 10,131 probes enriched in single-nucleotide polymorphisms (SNPs) were removed owing to the overlap of the final three bases in their sequences with those in the SNPs; 80,378 probes with > 10% missing values were also removed. This left a total of 391,918 probes and 885 samples in the final dataset (Fig. 2A and B).

We captured the distribution of methylation values for the differentially methylated sites according to an FDR adjusted P < 0.05 and an absolute mean methylation difference > 0.3 (Additional file 1: Table S1). Following extraction of methylation values at the gene level, we performed differential methylation analysis (Additional file 2: Table S2). We counted the distribution of methylation values for the differentially methylated genes in each group and generated a volcano plot of the differentially methylated genes (Fig. 2C and D). Figure 2E shows the 5775 differentially methylated sites as a heat map of the distribution between the two groups for further analysis.

### LncRNA markers identified according to differential methylation

We selected the 5775 differentially methylated sites and obtained 14,629 lncRNA expression profiles. By collapsing the methylation markers to genes, we paired the methylation sites with the lncRNAs to yield 1350 differentially methylated lncRNAs for subsequent analysis (Additional file 3: Table S3).

### Prognostic validation of LncRNAs demonstrating differential methylation

We then performed follow-up survival analysis using the 1350 differentially methylated lncRNAs. Removal of samples lacking survival information yielded a dataset with 1073 BC tumour samples, which was then divided into a training and a test set containing 537 and 536 samples, respectively. Survival analysis of 1350 lncRNAs from the 537 samples in the training set for survival identified 20 survival-related lncRNA markers. According to Akaike information criterion for the optimal combined model, 14 lncRNAs were ultimately selected as the prognostic model (MylnBrna). The optimal combinations and models were, as follows: ensg00000235576, ensg00000237248, ensg00000250971, ensg00000232352, ensg00000280241, ensg00000235840, ensg00000236859, ensg00000264589, ensg00000224509, ensg00000258077, ensg00000272463, ensg00000261215, ensg00000233723, and ensg00000224271. Table 1 provides details regarding this set of differentially methylated lncRNAs.

**Table 1:** The 14 potential lncRNA prognostic markers

ENSG ID	Position (CHR: Start–End)	Aliases	Strand
ENSG00000235576	Chromosome 2: 7,725,801–7,730,705	LINC01871	Forward
ENSG00000237248	Chromosome 12: 9,240,003–9,257,960	LINC00987	Forward
ENSG00000250971	Chromosome 4: 187,005,944–187,060,930	Lnc-F11-2	Forward
ENSG00000232352	Chromosome 3: 50,266,641–50,267,371	SEMA3B-AS1	Reverse
ENSG00000280241	Chromosome 4: 153,948,718–154,300,500	lnc-FGB-3	Forward
ENSG00000235840	Chromosome 2: 120,319,007–120,326,298	lnc-TMEM185B-6	Forward
ENSG00000236859	Chromosome 2: 121,649,320–121,728,563	NIFK-AS1	Forward
ENSG00000264589	Chromosome 17: 45,799,390–45,895,680	MAPT-AS1	Reverse
ENSG00000224509	Chromosome 2: 104,936,241–105,038,496	MRPS9-AS2	Reverse
ENSG00000258077	Chromosome 12: 75,563,202–75,984,015	lnc-GLIPR1-7	Reverse
ENSG00000272463	Chromosome 6: 708,592–711,405	lnc-IRF4-8	Reverse
ENSG00000261215	Chromosome 9: 34,661,903–34,666,029	lnc-IL11RA-2	Reverse
ENSG00000233723	Chromosome 2: 58,427,799–59,063,766	LINC01122	Forward
ENSG00000224271	Chromosome 22: 47,630,827–48,023,004	EPIC1	Forward

### Performance of the model in BC prognosis

**Fig. 3:**
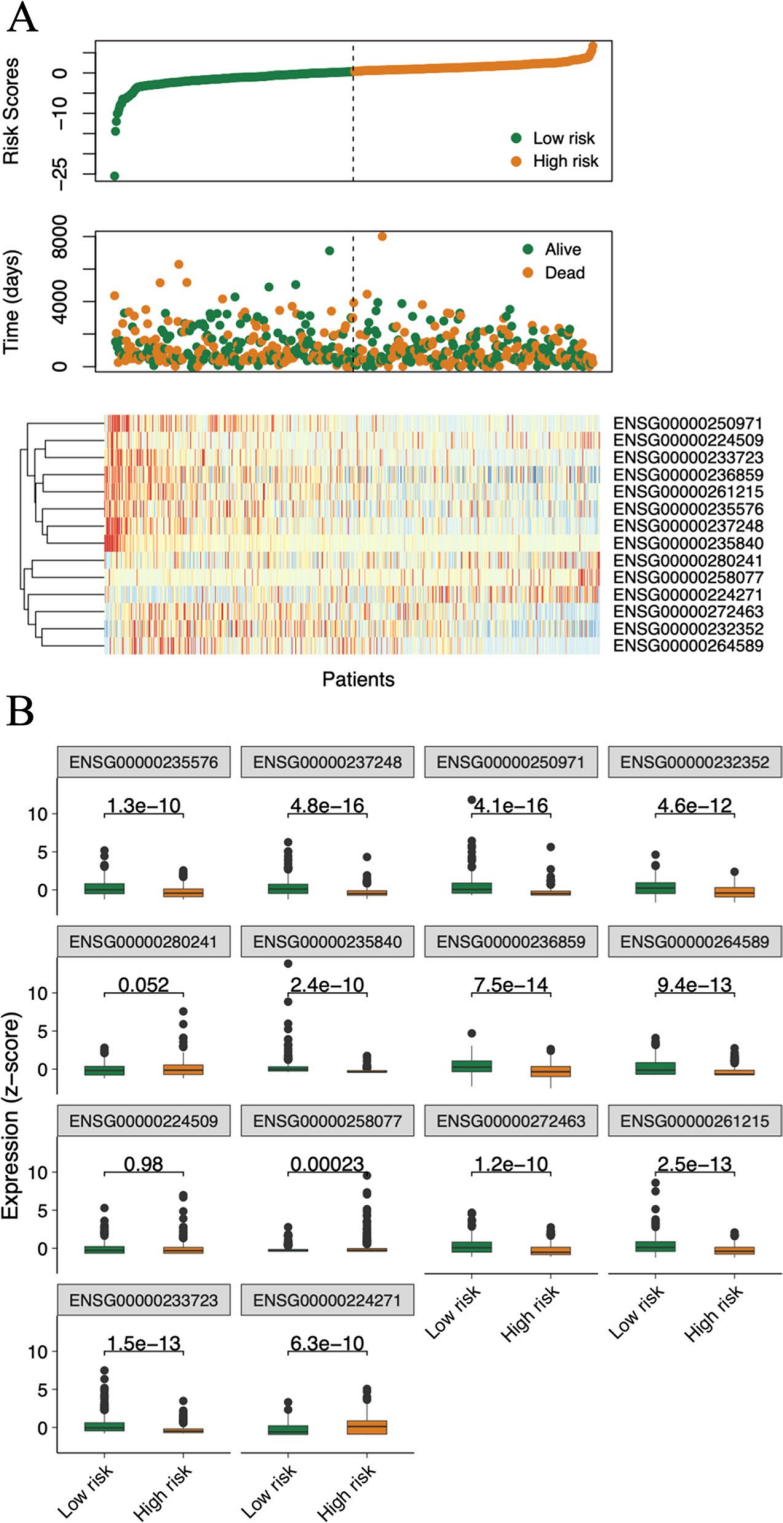
Performance of the MylnBrna model in BC prognosis. **A** Scatterplots of patient score and survival status distributions as well as a heat map of marker expression in the training set. **B** Box plots of the expression of four lncRNAs as potential prognostic markers in both high-risk and low-risk groups in the training set. BC, breast cancer

**Fig. 4:**
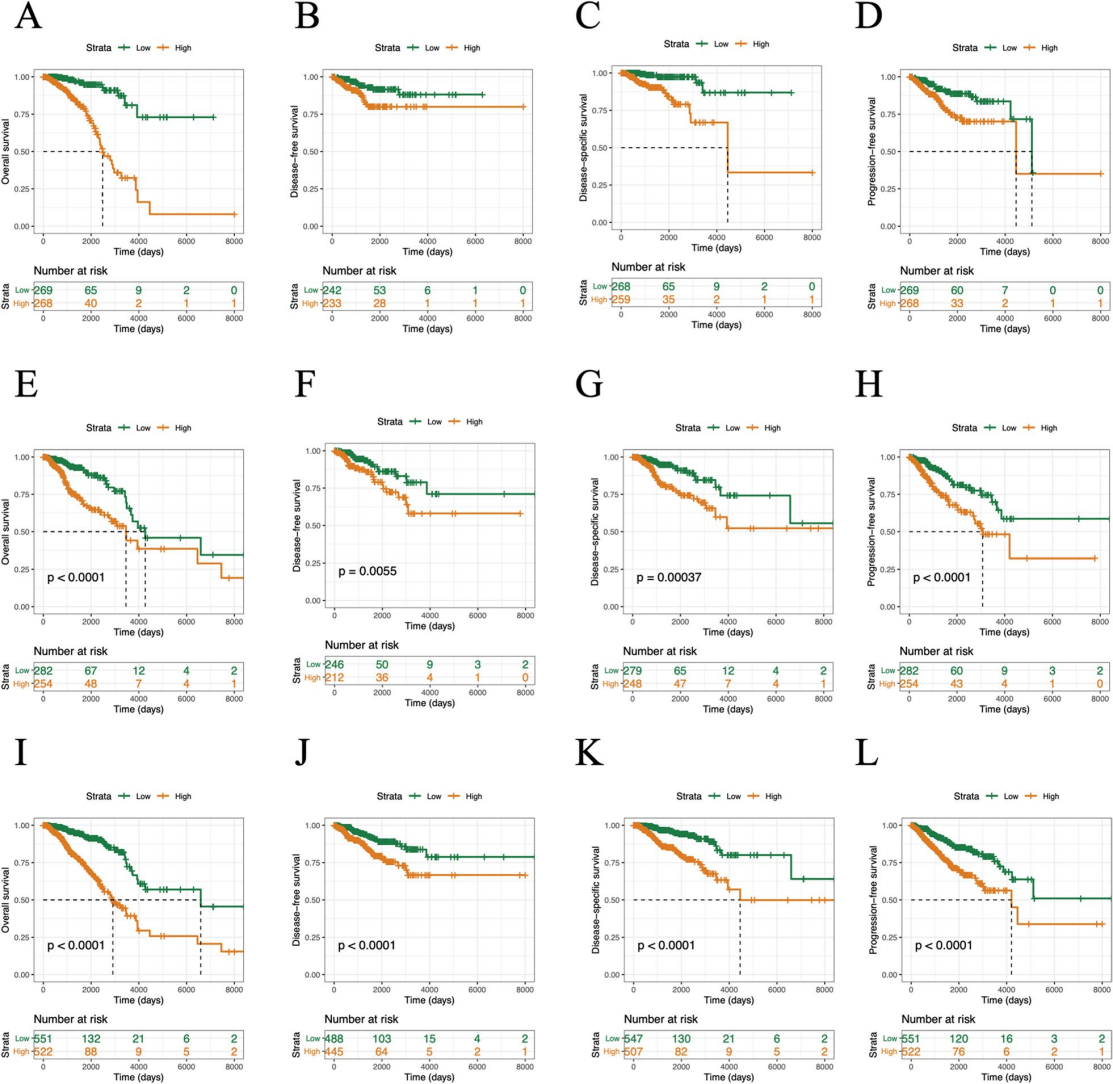
Survival curves over 3 and 5 years for low-risk and high-risk groups. Overall survival curves, disease-free survival curves, disease-specific survival curves, and progression-free survival curves generated using data from the (**A**–**D**) training set, (**E**–**H**) test set, and (**I**–**L**) overall dataset, respectively

To evaluate whether the performance of MylnBrna in BC prognosis was independent of other clinical features, we conducted univariate Cox regression (Additional file 4: Table S4) and multivariate Cox regression (Table 2) analyses for the individual clinical variables. Scatter plots were generated for the results of single-factor and multivariate Cox analyses for the score distributions and survival status (Fig. 3A), and a heat map showing marker expression in the patients from the training set was constructed (Fig. 3B). We analysed both the training sets (Fig. 4A–D) and test sets (Fig. 4E–H), as well as the overall dataset (Fig. 4I–L) and generated 3- and 5-year survival curves (i.e., OS, DFS, DSS, and PFS) to demonstrate the prognostic value of MylnBrna.

**Table 2:** The prognostic performance of the 14 lncRNA markers

lncRNA ID	HR (95% CI)*	P value
ENSG00000235576	0.54 (0.38, 0.78)	0.00092
ENSG00000237248	0.60 (0.41, 0.87)	0.0079
ENSG00000250971	0.73 (0.49, 1.07)	0.001
ENSG00000232352	0.57 (0.40, 0.81)	0.0015
ENSG00000280241	1.55 (1.28, 1.87)	4.70E-06
ENSG00000235840	0.17 (0.07, 0.42)	0.00011
ENSG00000236859	1.51 (1.12, 2.03)	0.0072
ENSG00000264589	0.56 (0.39, 0.79)	0.0013
ENSG00000224509	1.33 (1.09, 1.63)	0.0049
ENSG00000258077	1.49 (1.14, 1.95)	0.0037
ENSG00000272463	0.61 (0.43, 0.87)	0.0057
ENSG00000261215	0.75 (0.53, 1.05)	0.095
ENSG00000233723	0.57 (0.35, 0.94)	0.028
ENSG00000224271	1.51 (1.19, 1.90)	0.00054

^*^The hazard ratio ((HR) with 95% confidence interval (CI)) and P-value from the multivariate Cox regression analysis of each lncRNA are indicated

### Prognostic LncRNA markers and up-regulated tumour metabolic pathways

**Fig. 5:**
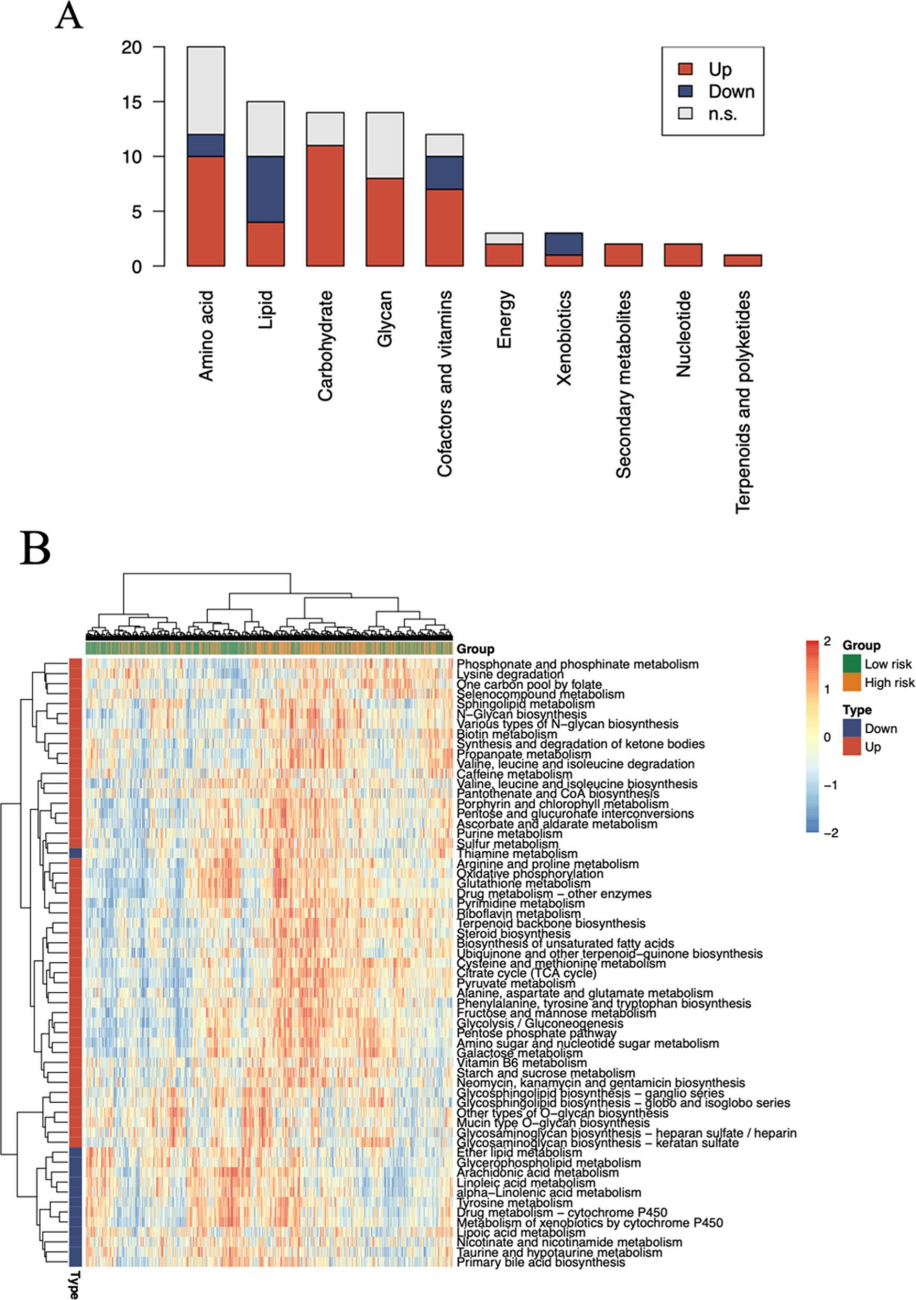
Prognostic lncRNA markers and upregulated tumour metabolic pathways. **A** Bar graph of significantly different metabolic pathways between the two groups showing high and low risk of survival. **B** Heat map showing activity of the 61 significantly up-regulated pathways between the high-risk and low-risk groups (pathway information acquired from Kyoto Encyclopaedia of Genes and Genomes database developed by Kanehisa Laboratories, https://www.kegg.jp/). n.s., no significant difference

To investigate the roles of the prognostic lncRNAs and their correlation with tumour metabolism, we performed association analysis. For the 10 major categories containing the 86 pathways (Additional file 5: Table S5), we determined whether the pathways within each category were significantly up-regulated or down-regulated in the high-risk group. Among the 14 potential lncRNA biomarkers, those related to amino acid metabolism pathways showed the highest up-regulation (Fig. 5A), followed by those related to lipid, carbohydrate, glycan, cofactor and vitamin, energy, xenobiotic, nucleotide, terpenoid, and polyketide metabolism, as well as secondary metabolites. A heat map of significant pathways was drawn from the 61 pathways with significantly elevated activity, among which amino acid metabolism was associated with prognostic lncRNAs modified by methylation, indicating a potential correlation between the 14 lncRNAs and tumour metabolism (Fig. 5B).

### Functional enrichment

**Fig. 6:**
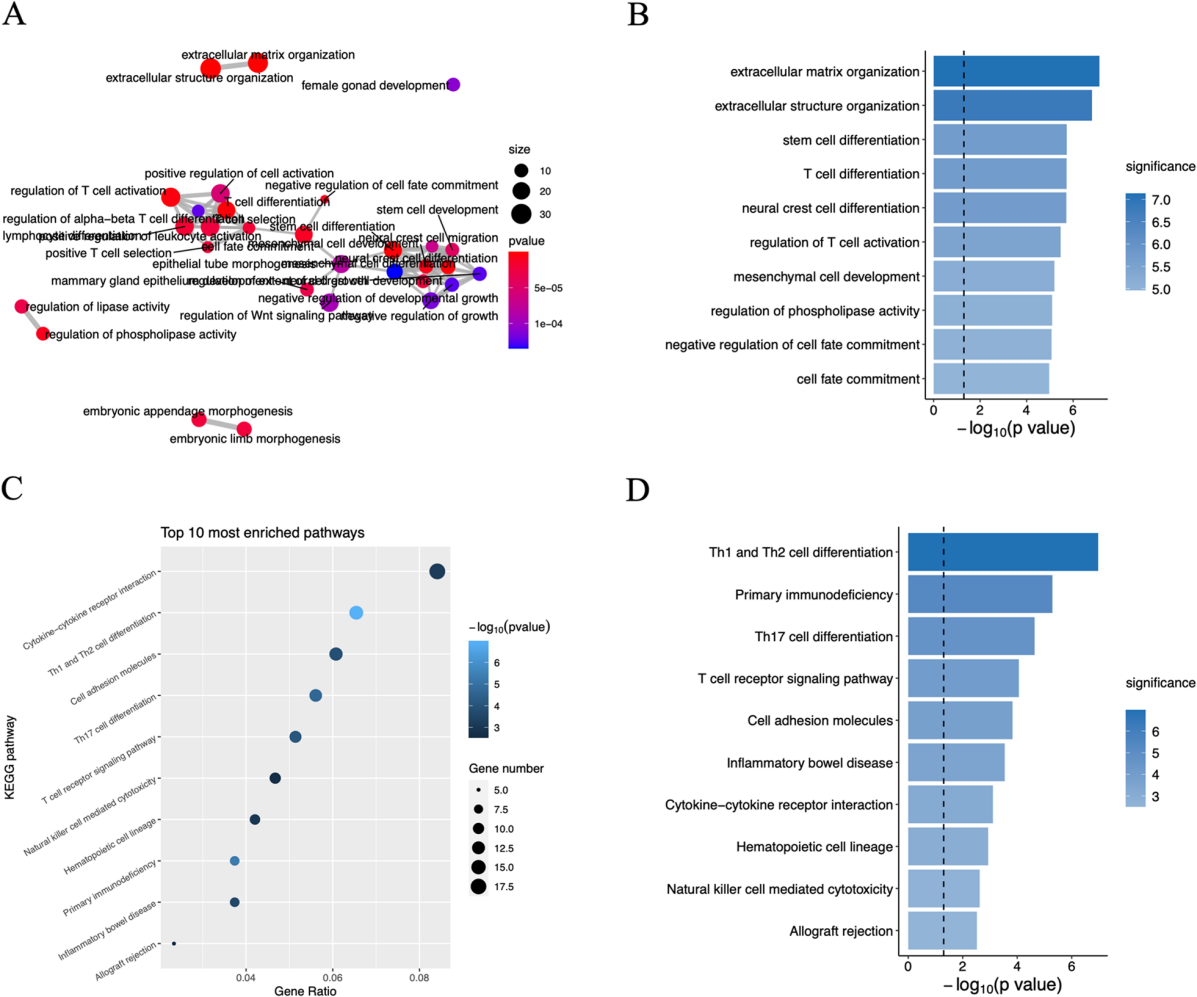
Functional enrichment analysis of MylnBrna. **A** Functional enrichment map of Gene Ontology (GO) results for the 14 lncRNA prognostic biomarkers. The top 50 from among 642 mRNAs were determined. **B** Ten GO terms showing the highest enrichment are shown. **C** Bubble chart for Kyoto Encyclopaedia of Genes and Genomes (KEGG) functional enrichment (pathway information acquired from KEGG database developed by Kanehisa Laboratories, https://www.kegg.jp/). **D** Ten pathways showing the highest enrichment are shown

To infer the potential biological roles of the identified lncRNAs, we calculated the Pearson’s correlation coefficient between their expression and those of the corresponding mRNAs. The top 50 mRNAs considered as lncRNA-related (Fig. 6A) were selected. Then we performed GO (Additional file 6: Table S6) and KEGG (Additional file 7: Table S7) functional enrichment analyses for 642 lncRNA-related mRNAs, among which 14 potential lncRNA prognostic biomarkers were functionally enriched in the GO database (Fig. 6A). We identified the top 10 KEGG pathways with the highest enrichment, with cytokine-receptor interaction being the most enriched pathway (Fig. 6B–D).

## Discussion

This study investigated the roles of differentially methylated lncRNAs in BC to determine possible biomarkers of altered BC tumour metabolism as well as prognostic markers of chemotherapy response. The analysis included 885 samples and 91,918 probes, resulting in the identification of 1350 differentially methylated lncRNAs for subsequent analysis. Screening identified 14 lncRNAs as prognosis-related markers for BC, with KEGG analysis of possibly altered metabolic pathways confirming that lncRNAs related to amino acid metabolism were significantly up-regulated in the high-risk BC group, suggesting the prognostic significance of these lncRNAs.

Although numerous lncRNAs are involved in BC development, treatment, and recurrence, few have been identified as playing a significant role [30, 31]. A previous study reported that up-regulated lncRNAs are associated with cyclin D1 binding and subsequent degradation, resulting in BC resistance to endocrine therapy and recurrence [4]. Other studies reported the effectiveness of targeted lncRNA therapy in animal experiments, suggesting the potential role of lncRNAs as prognostic biomarkers of drug resistance in patients with BC [4, 32]. For recurrent and refractory BCs resistant to chemotherapy and endocrine therapy, as well as triple-negative BCs, lncRNA-specific targeted therapies represent valuable and promising remedial treatment options based on their reported contribution to tumour progression [33–35].

Epigenetic modifications mediated by lncRNAs can lead to reprogramming of energy metabolism through complex and diverse pathways, including ubiquitination, phosphorylation, and acetylation. A recent study reported organelle-associated lncRNAs as potential clinical targets for manipulating cellular metabolism and disease, with mitochondria-localized lncRNAs identified as tumour suppressors favouring cellular energy homeostasis [36]. Additionally, previous studies indicate that epigenetically regulated lncRNAs may correlate with tumour metabolism, thus playing a vital role in enhancing tumour proliferation and progression and potentially resulting in BC resistance to treatment [37, 38]. Given the large amounts of data associated with gene regulation made available by advanced methods, the establishment of models capable of prognostic predictions related to treatment outcomes will promote subsequent research and clinical benefits.

The number of functional lncRNAs that have been well-studied for epigenetic regulation is relatively trivial compared to the numerous lncRNAs identified and documented in public databases. Among the 14 lncRNAs in MylnBrna, several lncRNAs have been well studied in several abnormal metabolic diseases and tumours. For instance, ensg00000237248 (LINC00987) has been reported to ameliorate chronic obstructive pulmonary disease through modulating lipopolysaccharide-induced cell apoptosis, oxidative stress, inflammation, and autophagy via regulating other gene signalling pathways [39]. The down-regulation of ensg00000232352 (lncRNA SEMA3B-AS1) was related to risky outcomes of patients with Wilms tumour [40]. Additionally, ensg00000236859 (lncRNA NIFK-AS1) was highly expressed in hepatic cancer tissues due to m6 methylation, and the knockdown of NIFK-AS1 sensitized tumour cells to sorafenib through upregulation of drug transport proteins [41]. This lncRNA NIFK-AS1 has also promoted the proliferation, migration and invasion of endometrial cancer cells by enhancing inhibition of M2-like polarization of macrophages through down-expression [42]. ensg00000264589 (lncRNA MAPT-AS1), present in the antisense strand of the promoter region of MAPT (microtubule-associated protein tau), was positively associated with improved patient survival [43]. To further understand the functional role of MylnBrna in BC, we performed a functional enrichment analysis of the genes encoding related proteins that epistemically regulated lncRNAs by considering their co-expression relationships and found that MylnBrna is associated with known tumour metabolic pathways, perhaps thereby serving as a cancer-related biological pathway.

Follow-up studies are required and planned. Although the significance of this field of research is widely recognized, relevant studies are limited, and existing knowledge has not been fully translated into clinical applications. Furthermore, given the scarcity of a large clinical database of BC-specific genes and lncRNA libraries available for exploration, the results of this study provide data for subsequent epigenetic studies of specific BCs, and the findings offer new perspectives on BC-specific tumour metabolism. The prognostic model of methylated lncRNAs in BC established in this study (MylnBrna) supports further evaluation and identification of biomarkers and therapeutic targets associated with tumour metabolism.

## Conclusion

A model of 14 lncRNAs that are associated with BC prognosis and that altered tumour metabolism was identified. Further studies are required to investigate other lncRNAs as potential metabolic biomarkers with predictive capacity for immunotherapeutic outcomes of patients with BC.

## Supplementary Information

**Additional file 1: Table S1. **Differentially methylated sites between cancer tissues and normal tissues for each gene site.

**Additional file 2: Table S2. **Differentially methylated genes between cancer tissues and normal tissues.

**Additional file 3:** **Table S3.** LncRNA–mRNA pairs associated with differentially methylated sites.

**Additional file 4: Table S4. **Univariate Cox analysis of 1350 differentially methylated lncRNAs.

**Additional file 5: Table S5. **The 86 KEGG pathways correlated with tumor metabolism.

**Additional file 6:** **Table S6. **GO biological processes related to the 14 potential lncRNA biomarkers.

**Additional file 7:** **Table S7. **KEGG pathways associated with the 14 potential lncRNA biomarkers.

## Data Availability

The datasets analysed during the current study and the supplementary material for this article are accessible from figshare (https://doi.org/10.6084/m9.figshare.19158620.v1 and https://doi.org/10.6084/m9.figshare.19158560.v1).

## Abbreviations

BC: Breast cancer
CI: Confidence interval
DFS: Disease-free survival
DSS: Disease-specific survival
FDR: False discovery rate
GO: Gene ontology
HR: Hazard ratio
KEGG: Kyoto encyclopedia of genes and genomes
lncRNA: Long noncoding RNA
OS: Overall survival
PFS: Progression-free survival
SNP: Single-nucleotide polymorphism
