# Supplementary material for: Identification and validation of an epigenetically regulated long noncoding RNA model for breast cancer metabolism and prognosis
Source: BMC Med Genomics. 2022 May 7;15:105. doi: 10.1186/s12920-022-01256-2 (PMC9077958; doi:10.1186/s12920-022-01256-2)
Supplement: Supplementary file 4 — Additional file 4: Table S4. Univariate Cox analysis of 1350 differentially methylated lncRNAs. [file 12920_2022_1256_MOESM4_ESM.zip › Supplementary_table_S4.docx]

**Supplementary table S4. Univariate cox analysis of 1350 lncRNAs associated with differential methylation-mediated prognosis.**

|  | HR | HR_lower | HR_upper | Z_score | P_value |
| --- | --- | --- | --- | --- | --- |
| ENSG00000242268 | 0.83 | 0.57 | 1.19 | -1.03 | 0.3 |
| ENSG00000235875 | 0.78 | 0.55 | 1.1 | -1.4 | 0.16 |
| ENSG00000258545 | 1.05 | 0.82 | 1.33 | 0.36 | 0.72 |
| ENSG00000249042 | 1.08 | 0.84 | 1.37 | 0.58 | 0.56 |
| ENSG00000230747 | 0.74 | 0.54 | 1.02 | -1.86 | 0.063 |
| ENSG00000256508 | 0.76 | 0.51 | 1.13 | -1.37 | 0.17 |
| ENSG00000258572 | 0.64 | 0.44 | 0.94 | -2.28 | 0.023 |
| ENSG00000279118 | 0.9 | 0.7 | 1.17 | -0.79 | 0.43 |
| ENSG00000277987 | 0.6 | 0.34 | 1.03 | -1.84 | 0.066 |
| ENSG00000230138 | 1.1 | 0.89 | 1.36 | 0.86 | 0.39 |
| ENSG00000267653 | 0.72 | 0.48 | 1.08 | -1.58 | 0.11 |
| ENSG00000274536 | 0.9 | 0.69 | 1.18 | -0.77 | 0.44 |
| ENSG00000273348 | 0.79 | 0.58 | 1.08 | -1.48 | 0.14 |
| ENSG00000279447 | 1.09 | 0.89 | 1.32 | 0.82 | 0.41 |
| ENSG00000226237 | 0.91 | 0.69 | 1.18 | -0.73 | 0.47 |
| ENSG00000267325 | 1.03 | 0.79 | 1.35 | 0.23 | 0.82 |
| ENSG00000259134 | 0.86 | 0.65 | 1.13 | -1.08 | 0.28 |
| ENSG00000265688 | 0.83 | 0.62 | 1.09 | -1.33 | 0.18 |
| ENSG00000272914 | 0.89 | 0.66 | 1.19 | -0.79 | 0.43 |
| ENSG00000225731 | 0.81 | 0.58 | 1.13 | -1.24 | 0.21 |
| ENSG00000261737 | 0.94 | 0.74 | 1.2 | -0.48 | 0.63 |
| ENSG00000255389 | 0.87 | 0.66 | 1.14 | -1.02 | 0.31 |
| ENSG00000238121 | 0.8 | 0.58 | 1.09 | -1.4 | 0.16 |
| ENSG00000232759 | 0.9 | 0.66 | 1.21 | -0.71 | 0.48 |
| ENSG00000179428 | 0.83 | 0.61 | 1.11 | -1.27 | 0.2 |
| ENSG00000253428 | 0.8 | 0.58 | 1.1 | -1.35 | 0.18 |
| ENSG00000267532 | 0.69 | 0.49 | 0.96 | -2.23 | 0.026 |
| ENSG00000281769 | 0.63 | 0.38 | 1.03 | -1.83 | 0.067 |
| ENSG00000226871 | 0.86 | 0.63 | 1.16 | -0.99 | 0.32 |
| ENSG00000254802 | 0.74 | 0.52 | 1.06 | -1.63 | 0.1 |
| ENSG00000272701 | 1.05 | 0.81 | 1.36 | 0.35 | 0.73 |
| ENSG00000236403 | 0.67 | 0.46 | 0.98 | -2.04 | 0.041 |
| ENSG00000245954 | 0.86 | 0.64 | 1.15 | -1.01 | 0.31 |
| ENSG00000245025 | 0.82 | 0.63 | 1.06 | -1.55 | 0.12 |
| ENSG00000248668 | 0.71 | 0.51 | 1.01 | -1.9 | 0.057 |
| ENSG00000228692 | 0.89 | 0.67 | 1.18 | -0.82 | 0.41 |
| ENSG00000243243 | 0.98 | 0.76 | 1.27 | -0.16 | 0.87 |
| ENSG00000246223 | 0.82 | 0.6 | 1.12 | -1.26 | 0.21 |
| ENSG00000274021 | 0.98 | 0.75 | 1.26 | -0.19 | 0.85 |
| ENSG00000267365 | 0.84 | 0.61 | 1.17 | -1.03 | 0.3 |
| ENSG00000259834 | 0.9 | 0.68 | 1.19 | -0.73 | 0.47 |
| ENSG00000229246 | 0.72 | 0.48 | 1.08 | -1.6 | 0.11 |
| ENSG00000251301 | 0.71 | 0.47 | 1.09 | -1.56 | 0.12 |
| ENSG00000277684 | 0.81 | 0.6 | 1.1 | -1.33 | 0.18 |
| ENSG00000277954 | 1.1 | 0.95 | 1.27 | 1.22 | 0.22 |
| ENSG00000280135 | 0.85 | 0.63 | 1.15 | -1.04 | 0.3 |
| ENSG00000261625 | 0.83 | 0.63 | 1.09 | -1.34 | 0.18 |
| ENSG00000235531 | 1.23 | 0.97 | 1.57 | 1.71 | 0.087 |
| ENSG00000259407 | 0.89 | 0.7 | 1.13 | -0.95 | 0.34 |
| ENSG00000273824 | 0.94 | 0.71 | 1.24 | -0.44 | 0.66 |
| ENSG00000250934 | 0.84 | 0.62 | 1.13 | -1.16 | 0.25 |
| ENSG00000253177 | 1.17 | 0.89 | 1.53 | 1.13 | 0.26 |
| ENSG00000241684 | 0.79 | 0.59 | 1.05 | -1.62 | 0.11 |
| ENSG00000247774 | 0.74 | 0.54 | 1 | -1.98 | 0.048 |
| ENSG00000230918 | 1.09 | 0.85 | 1.41 | 0.69 | 0.49 |
| ENSG00000232079 | 0.61 | 0.4 | 0.92 | -2.37 | 0.018 |
| ENSG00000234961 | 0.98 | 0.75 | 1.27 | -0.17 | 0.87 |
| ENSG00000241657 | 0.84 | 0.61 | 1.14 | -1.14 | 0.26 |
| ENSG00000260306 | 0.85 | 0.62 | 1.18 | -0.95 | 0.34 |
| ENSG00000233429 | 0.76 | 0.56 | 1.03 | -1.78 | 0.076 |
| ENSG00000230155 | 0.65 | 0.46 | 0.91 | -2.47 | 0.014 |
| ENSG00000223403 | 0.87 | 0.62 | 1.22 | -0.8 | 0.42 |
| ENSG00000228427 | 0.91 | 0.68 | 1.2 | -0.7 | 0.48 |
| ENSG00000246022 | 0.57 | 0.33 | 0.98 | -2.03 | 0.043 |
| ENSG00000228495 | 0.94 | 0.68 | 1.28 | -0.42 | 0.68 |
| ENSG00000254862 | 0.74 | 0.54 | 1.01 | -1.87 | 0.061 |
| ENSG00000229664 | 0.85 | 0.62 | 1.17 | -1.01 | 0.31 |
| ENSG00000231246 | 0.8 | 0.6 | 1.07 | -1.52 | 0.13 |
| ENSG00000259705 | 0.87 | 0.64 | 1.19 | -0.87 | 0.38 |
| ENSG00000245694 | 1.02 | 0.78 | 1.33 | 0.15 | 0.88 |
| ENSG00000273771 | 0.73 | 0.53 | 1.02 | -1.85 | 0.065 |
| ENSG00000227467 | 0.83 | 0.59 | 1.16 | -1.08 | 0.28 |
| ENSG00000177133 | 0.84 | 0.59 | 1.19 | -0.99 | 0.32 |
| ENSG00000256427 | 0.83 | 0.62 | 1.11 | -1.24 | 0.21 |
| ENSG00000243836 | 0.92 | 0.73 | 1.16 | -0.68 | 0.49 |
| ENSG00000241158 | 0.66 | 0.43 | 1.02 | -1.88 | 0.06 |
| ENSG00000281852 | 1 | 0.76 | 1.32 | 0 | 1 |
| ENSG00000277851 | 0.9 | 0.68 | 1.18 | -0.79 | 0.43 |
| ENSG00000255399 | 1.08 | 0.84 | 1.39 | 0.58 | 0.56 |
| ENSG00000186594 | 1.11 | 0.88 | 1.39 | 0.85 | 0.4 |
| ENSG00000234456 | 0.91 | 0.71 | 1.17 | -0.7 | 0.48 |
| ENSG00000267069 | 0.82 | 0.57 | 1.18 | -1.07 | 0.29 |
| ENSG00000263586 | 1.08 | 0.87 | 1.34 | 0.71 | 0.47 |
| ENSG00000267328 | 0.88 | 0.65 | 1.19 | -0.83 | 0.41 |
| ENSG00000263400 | 1.17 | 0.97 | 1.4 | 1.65 | 0.099 |
| ENSG00000262115 | 0.8 | 0.59 | 1.09 | -1.42 | 0.15 |
| ENSG00000280153 | 0.76 | 0.58 | 1 | -1.94 | 0.052 |
| ENSG00000276778 | 1.08 | 0.88 | 1.34 | 0.74 | 0.46 |
| ENSG00000237980 | 0.9 | 0.66 | 1.23 | -0.67 | 0.5 |
| ENSG00000226239 | 0.88 | 0.65 | 1.19 | -0.86 | 0.39 |
| ENSG00000235576 | 0.63 | 0.45 | 0.87 | -2.84 | 0.0046 |
| ENSG00000259004 | 0.83 | 0.63 | 1.09 | -1.33 | 0.18 |
| ENSG00000262097 | 0.87 | 0.67 | 1.14 | -0.99 | 0.32 |
| ENSG00000261586 | 1.09 | 0.87 | 1.36 | 0.72 | 0.47 |
| ENSG00000235366 | 0.93 | 0.71 | 1.22 | -0.55 | 0.58 |
| ENSG00000270412 | 0.91 | 0.7 | 1.2 | -0.64 | 0.52 |
| ENSG00000272789 | 0.84 | 0.63 | 1.13 | -1.12 | 0.26 |
| ENSG00000280143 | 0.91 | 0.71 | 1.17 | -0.73 | 0.46 |
| ENSG00000225434 | 1.09 | 0.84 | 1.42 | 0.63 | 0.53 |
| ENSG00000272282 | 0.76 | 0.55 | 1.06 | -1.64 | 0.1 |
| ENSG00000277879 | 0.77 | 0.57 | 1.04 | -1.69 | 0.091 |
| ENSG00000277631 | 0.72 | 0.5 | 1.05 | -1.71 | 0.087 |
| ENSG00000180139 | 0.83 | 0.63 | 1.09 | -1.35 | 0.18 |
| ENSG00000258810 | 0.91 | 0.7 | 1.19 | -0.67 | 0.5 |
| ENSG00000248441 | 0.92 | 0.7 | 1.21 | -0.57 | 0.57 |
| ENSG00000237484 | 0.95 | 0.74 | 1.22 | -0.4 | 0.69 |
| ENSG00000235904 | 0.95 | 0.72 | 1.26 | -0.33 | 0.74 |
| ENSG00000258168 | 1.02 | 0.8 | 1.29 | 0.12 | 0.9 |
| ENSG00000272783 | 1.06 | 0.82 | 1.38 | 0.45 | 0.65 |
| ENSG00000248309 | 0.85 | 0.63 | 1.14 | -1.1 | 0.27 |
| ENSG00000248008 | 0.91 | 0.71 | 1.17 | -0.74 | 0.46 |
| ENSG00000278727 | 0.99 | 0.79 | 1.24 | -0.1 | 0.92 |
| ENSG00000188525 | 0.89 | 0.67 | 1.19 | -0.77 | 0.44 |
| ENSG00000237499 | 0.85 | 0.64 | 1.12 | -1.15 | 0.25 |
| ENSG00000224318 | 0.94 | 0.73 | 1.21 | -0.47 | 0.64 |
| ENSG00000238057 | 0.75 | 0.54 | 1.04 | -1.72 | 0.085 |
| ENSG00000260947 | 0.97 | 0.77 | 1.24 | -0.22 | 0.82 |
| ENSG00000267074 | 0.83 | 0.62 | 1.12 | -1.22 | 0.22 |
| ENSG00000237125 | 0.88 | 0.67 | 1.17 | -0.87 | 0.38 |
| ENSG00000281103 | 0.77 | 0.57 | 1.05 | -1.65 | 0.1 |
| ENSG00000253552 | 0.74 | 0.55 | 1.02 | -1.86 | 0.063 |
| ENSG00000254211 | 0.76 | 0.52 | 1.11 | -1.42 | 0.16 |
| ENSG00000231943 | 0.63 | 0.42 | 0.96 | -2.16 | 0.03 |
| ENSG00000238120 | 1.02 | 0.8 | 1.31 | 0.18 | 0.86 |
| ENSG00000267122 | 0.67 | 0.46 | 0.96 | -2.17 | 0.03 |
| ENSG00000255471 | 0.97 | 0.75 | 1.27 | -0.2 | 0.84 |
| ENSG00000259436 | 0.76 | 0.54 | 1.08 | -1.54 | 0.12 |
| ENSG00000279204 | 0.89 | 0.67 | 1.19 | -0.76 | 0.45 |
| ENSG00000249669 | 0.8 | 0.59 | 1.1 | -1.36 | 0.17 |
| ENSG00000214548 | 0.9 | 0.67 | 1.2 | -0.73 | 0.47 |
| ENSG00000227508 | 0.9 | 0.65 | 1.25 | -0.64 | 0.52 |
| ENSG00000275234 | 0.97 | 0.74 | 1.27 | -0.24 | 0.81 |
| ENSG00000245164 | 0.87 | 0.64 | 1.16 | -0.95 | 0.34 |
| ENSG00000259347 | 0.74 | 0.48 | 1.13 | -1.38 | 0.17 |
| ENSG00000224958 | 0.81 | 0.6 | 1.09 | -1.41 | 0.16 |
| ENSG00000262370 | 0.77 | 0.58 | 1.04 | -1.69 | 0.09 |
| ENSG00000279036 | 0.78 | 0.54 | 1.12 | -1.35 | 0.18 |
| ENSG00000258733 | 0.84 | 0.61 | 1.14 | -1.14 | 0.26 |
| ENSG00000229484 | 1.05 | 0.8 | 1.38 | 0.37 | 0.71 |
| ENSG00000269220 | 0.86 | 0.64 | 1.16 | -0.99 | 0.32 |
| ENSG00000280339 | 0.92 | 0.71 | 1.18 | -0.67 | 0.51 |
| ENSG00000257219 | 1.01 | 0.78 | 1.31 | 0.08 | 0.94 |
| ENSG00000253434 | 0.81 | 0.57 | 1.17 | -1.13 | 0.26 |
| ENSG00000225399 | 1.05 | 0.8 | 1.38 | 0.38 | 0.7 |
| ENSG00000267107 | 0.9 | 0.68 | 1.18 | -0.77 | 0.44 |
| ENSG00000215386 | 0.86 | 0.67 | 1.12 | -1.1 | 0.27 |
| ENSG00000279086 | 1.08 | 0.82 | 1.43 | 0.55 | 0.58 |
| ENSG00000279019 | 1.16 | 0.88 | 1.52 | 1.05 | 0.29 |
| ENSG00000261438 | 0.84 | 0.65 | 1.09 | -1.29 | 0.2 |
| ENSG00000237248 | 0.59 | 0.41 | 0.87 | -2.7 | 0.0069 |
| ENSG00000271239 | 0.76 | 0.47 | 1.23 | -1.1 | 0.27 |
| ENSG00000261468 | 1.02 | 0.83 | 1.25 | 0.18 | 0.86 |
| ENSG00000279805 | 0.75 | 0.56 | 1 | -1.95 | 0.051 |
| ENSG00000236304 | 1.11 | 0.88 | 1.4 | 0.91 | 0.36 |
| ENSG00000229847 | 0.99 | 0.77 | 1.27 | -0.07 | 0.95 |
| ENSG00000260868 | 1.14 | 0.87 | 1.49 | 0.96 | 0.34 |
| ENSG00000232606 | 1.07 | 0.85 | 1.36 | 0.6 | 0.55 |
| ENSG00000275120 | 0.72 | 0.52 | 0.99 | -2.05 | 0.041 |
| ENSG00000267272 | 0.77 | 0.57 | 1.03 | -1.74 | 0.082 |
| ENSG00000225889 | 0.82 | 0.6 | 1.13 | -1.22 | 0.22 |
| ENSG00000231999 | 0.79 | 0.6 | 1.04 | -1.65 | 0.098 |
| ENSG00000228536 | 0.81 | 0.59 | 1.11 | -1.29 | 0.2 |
| ENSG00000261276 | 0.88 | 0.68 | 1.15 | -0.91 | 0.36 |
| ENSG00000250334 | 1.05 | 0.8 | 1.39 | 0.35 | 0.73 |
| ENSG00000236914 | 1 | 0.76 | 1.31 | -0.03 | 0.98 |
| ENSG00000274312 | 0.62 | 0.36 | 1.07 | -1.73 | 0.083 |
| ENSG00000250971 | 0.54 | 0.34 | 0.84 | -2.72 | 0.0064 |
| ENSG00000272411 | 0.97 | 0.75 | 1.25 | -0.24 | 0.81 |
| ENSG00000255248 | 0.89 | 0.69 | 1.14 | -0.95 | 0.34 |
| ENSG00000225670 | 0.85 | 0.63 | 1.14 | -1.1 | 0.27 |
| ENSG00000230587 | 0.83 | 0.6 | 1.14 | -1.17 | 0.24 |
| ENSG00000237813 | 0.84 | 0.61 | 1.16 | -1.06 | 0.29 |
| ENSG00000231858 | 0.89 | 0.67 | 1.19 | -0.77 | 0.44 |
| ENSG00000278214 | 0.88 | 0.64 | 1.22 | -0.76 | 0.44 |
| ENSG00000272823 | 0.86 | 0.66 | 1.12 | -1.12 | 0.26 |
| ENSG00000239636 | 0.75 | 0.54 | 1.04 | -1.73 | 0.083 |
| ENSG00000272482 | 1.01 | 0.79 | 1.31 | 0.11 | 0.91 |
| ENSG00000226252 | 0.96 | 0.74 | 1.24 | -0.3 | 0.76 |
| ENSG00000230435 | 1.01 | 0.84 | 1.2 | 0.06 | 0.95 |
| ENSG00000241163 | 0.81 | 0.57 | 1.16 | -1.14 | 0.25 |
| ENSG00000261269 | 0.99 | 0.78 | 1.26 | -0.07 | 0.95 |
| ENSG00000246430 | 1.04 | 0.79 | 1.37 | 0.27 | 0.78 |
| ENSG00000243961 | 0.85 | 0.6 | 1.2 | -0.92 | 0.36 |
| ENSG00000212719 | 1.16 | 0.92 | 1.46 | 1.28 | 0.2 |
| ENSG00000251257 | 1.09 | 0.85 | 1.4 | 0.66 | 0.51 |
| ENSG00000226197 | 1 | 0.76 | 1.3 | -0.03 | 0.98 |
| ENSG00000215483 | 0.92 | 0.68 | 1.25 | -0.53 | 0.59 |
| ENSG00000235172 | 0.83 | 0.63 | 1.1 | -1.29 | 0.2 |
| ENSG00000254703 | 0.82 | 0.62 | 1.09 | -1.39 | 0.16 |
| ENSG00000232821 | 1.13 | 0.96 | 1.33 | 1.5 | 0.13 |
| ENSG00000245812 | 0.95 | 0.71 | 1.26 | -0.39 | 0.7 |
| ENSG00000240143 | 0.92 | 0.71 | 1.18 | -0.66 | 0.51 |
| ENSG00000271856 | 0.92 | 0.69 | 1.21 | -0.62 | 0.53 |
| ENSG00000238078 | 1.01 | 0.78 | 1.31 | 0.1 | 0.92 |
| ENSG00000275088 | 0.91 | 0.66 | 1.24 | -0.6 | 0.55 |
| ENSG00000205562 | 1 | 0.77 | 1.3 | -0.01 | 0.99 |
| ENSG00000227039 | 0.82 | 0.61 | 1.09 | -1.37 | 0.17 |
| ENSG00000260102 | 0.92 | 0.67 | 1.27 | -0.51 | 0.61 |
| ENSG00000260721 | 0.88 | 0.61 | 1.28 | -0.66 | 0.51 |
| ENSG00000238018 | 0.79 | 0.58 | 1.07 | -1.52 | 0.13 |
| ENSG00000226482 | 0.83 | 0.51 | 1.36 | -0.73 | 0.47 |
| ENSG00000242258 | 1 | 0.77 | 1.29 | -0.02 | 0.98 |
| ENSG00000272941 | 0.78 | 0.58 | 1.05 | -1.62 | 0.1 |
| ENSG00000275025 | 1.08 | 0.93 | 1.26 | 1.02 | 0.31 |
| ENSG00000236849 | 0.97 | 0.77 | 1.23 | -0.22 | 0.82 |
| ENSG00000271334 | 0.8 | 0.58 | 1.1 | -1.39 | 0.17 |
| ENSG00000228649 | 0.8 | 0.61 | 1.04 | -1.69 | 0.091 |
| ENSG00000279711 | 1.02 | 0.74 | 1.42 | 0.14 | 0.89 |
| ENSG00000233093 | 0.89 | 0.66 | 1.2 | -0.76 | 0.45 |
| ENSG00000226031 | 0.94 | 0.79 | 1.13 | -0.65 | 0.52 |
| ENSG00000229108 | 0.77 | 0.55 | 1.07 | -1.54 | 0.12 |
| ENSG00000227260 | 1 | 0.81 | 1.24 | 0.04 | 0.97 |
| ENSG00000278058 | 1 | 0.78 | 1.29 | -0.01 | 0.99 |
| ENSG00000204261 | 0.77 | 0.59 | 1 | -1.93 | 0.053 |
| ENSG00000188511 | 0.96 | 0.71 | 1.3 | -0.25 | 0.8 |
| ENSG00000268001 | 1.04 | 0.81 | 1.35 | 0.32 | 0.75 |
| ENSG00000237513 | 0.79 | 0.56 | 1.12 | -1.32 | 0.19 |
| ENSG00000236333 | 0.7 | 0.44 | 1.12 | -1.48 | 0.14 |
| ENSG00000267121 | 0.72 | 0.52 | 1 | -1.97 | 0.049 |
| ENSG00000228401 | 0.83 | 0.64 | 1.07 | -1.43 | 0.15 |
| ENSG00000267364 | 0.85 | 0.65 | 1.1 | -1.23 | 0.22 |
| ENSG00000261472 | 0.96 | 0.68 | 1.35 | -0.25 | 0.81 |
| ENSG00000268027 | 0.83 | 0.61 | 1.13 | -1.19 | 0.23 |
| ENSG00000280304 | 1.06 | 0.85 | 1.33 | 0.52 | 0.6 |
| ENSG00000227591 | 0.79 | 0.59 | 1.06 | -1.6 | 0.11 |
| ENSG00000264016 | 1.19 | 1.02 | 1.39 | 2.17 | 0.03 |
| ENSG00000277763 | 0.88 | 0.68 | 1.13 | -0.99 | 0.32 |
| ENSG00000103472 | 0.94 | 0.73 | 1.21 | -0.48 | 0.63 |
| ENSG00000225938 | 0.78 | 0.58 | 1.03 | -1.74 | 0.082 |
| ENSG00000226808 | 0.92 | 0.7 | 1.2 | -0.62 | 0.54 |
| ENSG00000224525 | 0.73 | 0.47 | 1.12 | -1.44 | 0.15 |
| ENSG00000248869 | 0.98 | 0.68 | 1.4 | -0.12 | 0.91 |
| ENSG00000237803 | 1.04 | 0.76 | 1.42 | 0.24 | 0.81 |
| ENSG00000254231 | 0.94 | 0.73 | 1.21 | -0.5 | 0.62 |
| ENSG00000231367 | 0.74 | 0.54 | 1.01 | -1.89 | 0.059 |
| ENSG00000260710 | 0.89 | 0.68 | 1.16 | -0.88 | 0.38 |
| ENSG00000261616 | 0.81 | 0.6 | 1.09 | -1.37 | 0.17 |
| ENSG00000197308 | 0.81 | 0.63 | 1.05 | -1.56 | 0.12 |
| ENSG00000269918 | 0.78 | 0.59 | 1.02 | -1.83 | 0.067 |
| ENSG00000231826 | 0.98 | 0.78 | 1.23 | -0.21 | 0.84 |
| ENSG00000229116 | 0.92 | 0.66 | 1.26 | -0.54 | 0.59 |
| ENSG00000226419 | 0.9 | 0.69 | 1.19 | -0.74 | 0.46 |
| ENSG00000234949 | 0.91 | 0.7 | 1.18 | -0.72 | 0.47 |
| ENSG00000237943 | 0.95 | 0.73 | 1.23 | -0.39 | 0.7 |
| ENSG00000266036 | 1.01 | 0.79 | 1.29 | 0.06 | 0.96 |
| ENSG00000281655 | 0.69 | 0.49 | 0.97 | -2.15 | 0.031 |
| ENSG00000228639 | 0.82 | 0.6 | 1.11 | -1.28 | 0.2 |
| ENSG00000238045 | 0.88 | 0.68 | 1.14 | -0.96 | 0.34 |
| ENSG00000236166 | 0.77 | 0.51 | 1.14 | -1.31 | 0.19 |
| ENSG00000280294 | 0.91 | 0.69 | 1.2 | -0.67 | 0.5 |
| ENSG00000259583 | 0.94 | 0.73 | 1.21 | -0.49 | 0.62 |
| ENSG00000274653 | 0.78 | 0.59 | 1.03 | -1.77 | 0.076 |
| ENSG00000226409 | 0.99 | 0.77 | 1.26 | -0.12 | 0.91 |
| ENSG00000270069 | 0.89 | 0.66 | 1.2 | -0.77 | 0.44 |
| ENSG00000246090 | 0.76 | 0.57 | 1.03 | -1.75 | 0.081 |
| ENSG00000225194 | 0.88 | 0.64 | 1.19 | -0.85 | 0.4 |
| ENSG00000245904 | 0.77 | 0.54 | 1.1 | -1.42 | 0.15 |
| ENSG00000235997 | 0.92 | 0.71 | 1.19 | -0.65 | 0.51 |
| ENSG00000278948 | 0.87 | 0.66 | 1.14 | -1.02 | 0.31 |
| ENSG00000228223 | 1.05 | 0.83 | 1.33 | 0.41 | 0.68 |
| ENSG00000270177 | 0.92 | 0.71 | 1.2 | -0.61 | 0.54 |
| ENSG00000261175 | 0.88 | 0.66 | 1.17 | -0.86 | 0.39 |
| ENSG00000260219 | 0.88 | 0.67 | 1.17 | -0.87 | 0.38 |
| ENSG00000153363 | 0.88 | 0.68 | 1.14 | -0.96 | 0.34 |
| ENSG00000271155 | 0.9 | 0.68 | 1.19 | -0.74 | 0.46 |
| ENSG00000237989 | 0.83 | 0.6 | 1.13 | -1.18 | 0.24 |
| ENSG00000223813 | 0.93 | 0.72 | 1.19 | -0.62 | 0.54 |
| ENSG00000274173 | 1 | 0.77 | 1.3 | -0.02 | 0.98 |
| ENSG00000256916 | 0.73 | 0.53 | 1.01 | -1.91 | 0.056 |
| ENSG00000281392 | 0.8 | 0.6 | 1.08 | -1.45 | 0.15 |
| ENSG00000226005 | 0.73 | 0.51 | 1.03 | -1.79 | 0.074 |
| ENSG00000226833 | 0.76 | 0.57 | 1.02 | -1.83 | 0.067 |
| ENSG00000261054 | 0.98 | 0.78 | 1.24 | -0.13 | 0.89 |
| ENSG00000170919 | 0.71 | 0.52 | 0.98 | -2.09 | 0.037 |
| ENSG00000231638 | 0.87 | 0.64 | 1.18 | -0.9 | 0.37 |
| ENSG00000270547 | 1.26 | 1.05 | 1.52 | 2.45 | 0.014 |
| ENSG00000231768 | 0.99 | 0.76 | 1.3 | -0.05 | 0.96 |
| ENSG00000231290 | 1.03 | 0.83 | 1.27 | 0.23 | 0.82 |
| ENSG00000228971 | 0.85 | 0.64 | 1.14 | -1.1 | 0.27 |
| ENSG00000254847 | 0.82 | 0.63 | 1.09 | -1.38 | 0.17 |
| ENSG00000254510 | 0.86 | 0.63 | 1.17 | -0.98 | 0.33 |
| ENSG00000250961 | 1.1 | 0.92 | 1.32 | 1.03 | 0.3 |
| ENSG00000279587 | 0.58 | 0.37 | 0.91 | -2.38 | 0.018 |
| ENSG00000260461 | 1.01 | 0.79 | 1.28 | 0.06 | 0.95 |
| ENSG00000278962 | 0.93 | 0.72 | 1.19 | -0.6 | 0.55 |
| ENSG00000180769 | 1.07 | 0.85 | 1.34 | 0.54 | 0.59 |
| ENSG00000223776 | 0.97 | 0.73 | 1.29 | -0.21 | 0.83 |
| ENSG00000225472 | 1.15 | 0.9 | 1.48 | 1.12 | 0.26 |
| ENSG00000267751 | 0.92 | 0.71 | 1.2 | -0.61 | 0.54 |
| ENSG00000269867 | 0.84 | 0.65 | 1.08 | -1.37 | 0.17 |
| ENSG00000228613 | 1 | 0.78 | 1.29 | 0.02 | 0.99 |
| ENSG00000224577 | 0.78 | 0.6 | 1 | -1.95 | 0.051 |
| ENSG00000205913 | 0.72 | 0.53 | 0.97 | -2.14 | 0.033 |
| ENSG00000234899 | 0.89 | 0.65 | 1.21 | -0.75 | 0.45 |
| ENSG00000263011 | 0.83 | 0.63 | 1.09 | -1.34 | 0.18 |
| ENSG00000229558 | 0.92 | 0.72 | 1.17 | -0.7 | 0.48 |
| ENSG00000238099 | 0.74 | 0.52 | 1.06 | -1.65 | 0.098 |
| ENSG00000276668 | 0.76 | 0.55 | 1.06 | -1.6 | 0.11 |
| ENSG00000268798 | 0.99 | 0.78 | 1.26 | -0.08 | 0.94 |
| ENSG00000270076 | 0.85 | 0.63 | 1.13 | -1.11 | 0.27 |
| ENSG00000271880 | 1.06 | 0.83 | 1.35 | 0.47 | 0.64 |
| ENSG00000269124 | 0.97 | 0.74 | 1.27 | -0.2 | 0.84 |
| ENSG00000278934 | 0.9 | 0.69 | 1.18 | -0.78 | 0.43 |
| ENSG00000265399 | 0.9 | 0.67 | 1.21 | -0.69 | 0.49 |
| ENSG00000269736 | 0.83 | 0.56 | 1.24 | -0.91 | 0.36 |
| ENSG00000259976 | 0.8 | 0.63 | 1.02 | -1.83 | 0.067 |
| ENSG00000225039 | 0.8 | 0.6 | 1.07 | -1.53 | 0.12 |
| ENSG00000280302 | 0.93 | 0.69 | 1.24 | -0.49 | 0.62 |
| ENSG00000235033 | 0.92 | 0.68 | 1.26 | -0.5 | 0.62 |
| ENSG00000228162 | 0.68 | 0.44 | 1.05 | -1.75 | 0.079 |
| ENSG00000256124 | 0.83 | 0.59 | 1.17 | -1.07 | 0.29 |
| ENSG00000279806 | 0.84 | 0.63 | 1.11 | -1.25 | 0.21 |
| ENSG00000255850 | 0.93 | 0.71 | 1.21 | -0.55 | 0.58 |
| ENSG00000258604 | 0.78 | 0.54 | 1.13 | -1.29 | 0.2 |
| ENSG00000259969 | 0.9 | 0.69 | 1.16 | -0.82 | 0.41 |
| ENSG00000277270 | 0.84 | 0.59 | 1.22 | -0.91 | 0.36 |
| ENSG00000254109 | 0.95 | 0.72 | 1.25 | -0.36 | 0.72 |
| ENSG00000264345 | 0.93 | 0.75 | 1.16 | -0.66 | 0.51 |
| ENSG00000255648 | 0.72 | 0.49 | 1.07 | -1.64 | 0.1 |
| ENSG00000232284 | 0.79 | 0.6 | 1.04 | -1.7 | 0.089 |
| ENSG00000229619 | 0.93 | 0.7 | 1.25 | -0.47 | 0.64 |
| ENSG00000260025 | 0.71 | 0.55 | 0.92 | -2.63 | 0.0086 |
| ENSG00000258376 | 0.8 | 0.61 | 1.04 | -1.64 | 0.1 |
| ENSG00000250343 | 0.81 | 0.51 | 1.27 | -0.94 | 0.35 |
| ENSG00000229312 | 0.83 | 0.58 | 1.17 | -1.08 | 0.28 |
| ENSG00000224307 | 0.98 | 0.74 | 1.3 | -0.13 | 0.89 |
| ENSG00000280362 | 0.56 | 0.35 | 0.91 | -2.35 | 0.019 |
| ENSG00000259920 | 1.17 | 0.93 | 1.47 | 1.3 | 0.19 |
| ENSG00000251448 | 0.93 | 0.71 | 1.21 | -0.53 | 0.6 |
| ENSG00000272143 | 0.7 | 0.53 | 0.92 | -2.52 | 0.012 |
| ENSG00000259604 | 0.92 | 0.67 | 1.24 | -0.57 | 0.57 |
| ENSG00000237560 | 0.87 | 0.58 | 1.3 | -0.67 | 0.5 |
| ENSG00000234471 | 0.77 | 0.5 | 1.2 | -1.15 | 0.25 |
| ENSG00000255216 | 0.75 | 0.47 | 1.22 | -1.16 | 0.25 |
| ENSG00000259744 | 1.07 | 0.87 | 1.3 | 0.62 | 0.54 |
| ENSG00000254951 | 0.72 | 0.5 | 1.04 | -1.74 | 0.082 |
| ENSG00000255310 | 0.84 | 0.65 | 1.1 | -1.27 | 0.2 |
| ENSG00000275612 | 0.76 | 0.55 | 1.05 | -1.67 | 0.095 |
| ENSG00000258942 | 0.92 | 0.68 | 1.23 | -0.59 | 0.56 |
| ENSG00000265374 | 0.86 | 0.62 | 1.17 | -0.97 | 0.33 |
| ENSG00000228528 | 0.82 | 0.6 | 1.12 | -1.24 | 0.21 |
| ENSG00000266601 | 0.77 | 0.59 | 1.02 | -1.85 | 0.064 |
| ENSG00000280429 | 0.67 | 0.44 | 1.03 | -1.81 | 0.071 |
| ENSG00000250602 | 0.87 | 0.66 | 1.14 | -1.01 | 0.31 |
| ENSG00000272777 | 0.8 | 0.61 | 1.05 | -1.63 | 0.1 |
| ENSG00000279267 | 0.93 | 0.72 | 1.2 | -0.57 | 0.57 |
| ENSG00000250538 | 0.63 | 0.34 | 1.16 | -1.49 | 0.14 |
| ENSG00000271967 | 0.74 | 0.56 | 0.97 | -2.16 | 0.031 |
| ENSG00000237949 | 0.89 | 0.65 | 1.22 | -0.72 | 0.47 |
| ENSG00000263508 | 1.03 | 0.85 | 1.25 | 0.34 | 0.73 |
| ENSG00000232444 | 0.85 | 0.65 | 1.13 | -1.11 | 0.27 |
| ENSG00000257766 | 0.69 | 0.42 | 1.14 | -1.43 | 0.15 |
| ENSG00000258655 | 0.83 | 0.63 | 1.1 | -1.32 | 0.19 |
| ENSG00000255191 | 0.69 | 0.49 | 0.96 | -2.22 | 0.026 |
| ENSG00000240423 | 0.92 | 0.7 | 1.19 | -0.66 | 0.51 |
| ENSG00000204528 | 0.85 | 0.6 | 1.22 | -0.87 | 0.38 |
| ENSG00000255774 | 0.84 | 0.64 | 1.09 | -1.29 | 0.2 |
| ENSG00000254814 | 1.21 | 0.96 | 1.52 | 1.65 | 0.1 |
| ENSG00000227719 | 0.75 | 0.57 | 0.97 | -2.2 | 0.027 |
| ENSG00000272335 | 0.85 | 0.66 | 1.09 | -1.27 | 0.2 |
| ENSG00000253164 | 0.97 | 0.73 | 1.3 | -0.19 | 0.85 |
| ENSG00000271916 | 0.79 | 0.62 | 1.02 | -1.79 | 0.074 |
| ENSG00000272269 | 0.76 | 0.55 | 1.05 | -1.66 | 0.096 |
| ENSG00000250740 | 0.87 | 0.67 | 1.13 | -1.03 | 0.3 |
| ENSG00000231566 | 1.13 | 0.86 | 1.49 | 0.87 | 0.38 |
| ENSG00000272654 | 0.82 | 0.63 | 1.06 | -1.54 | 0.12 |
| ENSG00000236740 | 1.17 | 0.92 | 1.48 | 1.3 | 0.19 |
| ENSG00000258623 | 0.97 | 0.7 | 1.34 | -0.17 | 0.87 |
| ENSG00000255750 | 0.62 | 0.36 | 1.07 | -1.73 | 0.083 |
| ENSG00000263335 | 0.85 | 0.65 | 1.1 | -1.26 | 0.21 |
| ENSG00000266441 | 0.9 | 0.66 | 1.23 | -0.65 | 0.51 |
| ENSG00000245750 | 1.05 | 0.81 | 1.37 | 0.38 | 0.7 |
| ENSG00000197180 | 0.89 | 0.68 | 1.17 | -0.81 | 0.42 |
| ENSG00000229162 | 1.11 | 0.85 | 1.45 | 0.77 | 0.44 |
| ENSG00000249684 | 1 | 0.78 | 1.29 | 0.02 | 0.99 |
| ENSG00000196810 | 0.87 | 0.68 | 1.1 | -1.17 | 0.24 |
| ENSG00000225265 | 0.97 | 0.75 | 1.25 | -0.27 | 0.79 |
| ENSG00000224687 | 1.06 | 0.83 | 1.35 | 0.46 | 0.65 |
| ENSG00000226953 | 0.94 | 0.71 | 1.23 | -0.45 | 0.65 |
| ENSG00000271850 | 0.99 | 0.78 | 1.25 | -0.11 | 0.91 |
| ENSG00000271826 | 1 | 0.73 | 1.35 | -0.03 | 0.98 |
| ENSG00000230082 | 0.94 | 0.71 | 1.23 | -0.46 | 0.64 |
| ENSG00000253348 | 0.96 | 0.73 | 1.27 | -0.26 | 0.79 |
| ENSG00000278133 | 0.88 | 0.68 | 1.15 | -0.95 | 0.34 |
| ENSG00000233593 | 0.8 | 0.59 | 1.08 | -1.45 | 0.15 |
| ENSG00000229525 | 0.93 | 0.72 | 1.2 | -0.57 | 0.57 |
| ENSG00000254607 | 1 | 0.78 | 1.28 | -0.01 | 0.99 |
| ENSG00000248429 | 0.98 | 0.75 | 1.27 | -0.19 | 0.85 |
| ENSG00000276649 | 1.01 | 0.79 | 1.29 | 0.08 | 0.93 |
| ENSG00000278928 | 1.16 | 0.96 | 1.4 | 1.56 | 0.12 |
| ENSG00000232692 | 0.91 | 0.7 | 1.18 | -0.7 | 0.48 |
| ENSG00000179082 | 0.96 | 0.73 | 1.26 | -0.32 | 0.75 |
| ENSG00000275223 | 1.19 | 0.93 | 1.51 | 1.4 | 0.16 |
| ENSG00000262772 | 0.87 | 0.66 | 1.15 | -0.97 | 0.33 |
| ENSG00000248360 | 0.92 | 0.72 | 1.17 | -0.67 | 0.51 |
| ENSG00000277310 | 0.85 | 0.65 | 1.12 | -1.16 | 0.24 |
| ENSG00000255007 | 1.06 | 0.83 | 1.36 | 0.48 | 0.63 |
| ENSG00000222044 | 0.8 | 0.49 | 1.31 | -0.88 | 0.38 |
| ENSG00000272323 | 0.94 | 0.71 | 1.23 | -0.48 | 0.63 |
| ENSG00000273107 | 0.96 | 0.74 | 1.23 | -0.34 | 0.73 |
| ENSG00000243384 | 1.02 | 0.81 | 1.28 | 0.15 | 0.88 |
| ENSG00000238279 | 1.05 | 0.82 | 1.33 | 0.36 | 0.72 |
| ENSG00000231106 | 0.98 | 0.77 | 1.27 | -0.12 | 0.91 |
| ENSG00000277829 | 0.83 | 0.62 | 1.11 | -1.24 | 0.22 |
| ENSG00000267480 | 0.98 | 0.75 | 1.28 | -0.17 | 0.86 |
| ENSG00000273901 | 0.82 | 0.61 | 1.08 | -1.41 | 0.16 |
| ENSG00000269210 | 0.84 | 0.59 | 1.2 | -0.96 | 0.34 |
| ENSG00000257060 | 0.7 | 0.45 | 1.1 | -1.55 | 0.12 |
| ENSG00000278192 | 0.81 | 0.58 | 1.14 | -1.2 | 0.23 |
| ENSG00000273472 | 0.82 | 0.63 | 1.07 | -1.47 | 0.14 |
| ENSG00000253522 | 0.9 | 0.69 | 1.15 | -0.85 | 0.39 |
| ENSG00000250620 | 1.09 | 0.85 | 1.39 | 0.69 | 0.49 |
| ENSG00000265096 | 1.05 | 0.82 | 1.34 | 0.36 | 0.72 |
| ENSG00000263105 | 0.7 | 0.51 | 0.95 | -2.29 | 0.022 |
| ENSG00000243629 | 0.85 | 0.6 | 1.21 | -0.92 | 0.36 |
| ENSG00000272913 | 1.08 | 0.83 | 1.41 | 0.57 | 0.57 |
| ENSG00000280594 | 1.01 | 0.73 | 1.39 | 0.05 | 0.96 |
| ENSG00000268204 | 0.77 | 0.58 | 1.03 | -1.77 | 0.076 |
| ENSG00000224559 | 0.81 | 0.62 | 1.05 | -1.63 | 0.1 |
| ENSG00000254815 | 0.69 | 0.51 | 0.95 | -2.3 | 0.022 |
| ENSG00000260810 | 0.8 | 0.61 | 1.06 | -1.57 | 0.12 |
| ENSG00000255367 | 1.06 | 0.84 | 1.34 | 0.46 | 0.64 |
| ENSG00000273387 | 0.91 | 0.68 | 1.22 | -0.62 | 0.54 |
| ENSG00000226137 | 0.74 | 0.58 | 0.94 | -2.48 | 0.013 |
| ENSG00000203688 | 0.89 | 0.65 | 1.21 | -0.75 | 0.45 |
| ENSG00000261664 | 0.87 | 0.68 | 1.11 | -1.12 | 0.26 |
| ENSG00000272825 | 1.09 | 0.87 | 1.36 | 0.77 | 0.44 |
| ENSG00000255202 | 0.8 | 0.57 | 1.11 | -1.33 | 0.18 |
| ENSG00000275763 | 0.97 | 0.77 | 1.23 | -0.22 | 0.83 |
| ENSG00000232325 | 1.02 | 0.79 | 1.32 | 0.15 | 0.88 |
| ENSG00000273203 | 0.91 | 0.7 | 1.18 | -0.72 | 0.47 |
| ENSG00000257271 | 0.95 | 0.72 | 1.25 | -0.36 | 0.72 |
| ENSG00000235009 | 0.91 | 0.67 | 1.23 | -0.62 | 0.54 |
| ENSG00000203808 | 0.85 | 0.6 | 1.21 | -0.91 | 0.36 |
| ENSG00000240859 | 0.96 | 0.75 | 1.23 | -0.35 | 0.73 |
| ENSG00000230393 | 0.89 | 0.68 | 1.16 | -0.87 | 0.38 |
| ENSG00000272192 | 0.85 | 0.63 | 1.14 | -1.1 | 0.27 |
| ENSG00000273001 | 0.93 | 0.73 | 1.2 | -0.54 | 0.59 |
| ENSG00000203635 | 1.01 | 0.78 | 1.3 | 0.07 | 0.94 |
| ENSG00000239332 | 1.04 | 0.79 | 1.35 | 0.25 | 0.8 |
| ENSG00000256128 | 0.83 | 0.59 | 1.16 | -1.1 | 0.27 |
| ENSG00000240207 | 0.82 | 0.63 | 1.07 | -1.49 | 0.14 |
| ENSG00000235192 | 0.96 | 0.73 | 1.25 | -0.31 | 0.76 |
| ENSG00000279930 | 0.69 | 0.49 | 0.95 | -2.26 | 0.024 |
| ENSG00000146521 | 0.88 | 0.66 | 1.16 | -0.94 | 0.35 |
| ENSG00000228436 | 0.98 | 0.74 | 1.3 | -0.13 | 0.89 |
| ENSG00000260807 | 1.03 | 0.81 | 1.3 | 0.21 | 0.83 |
| ENSG00000215256 | 0.87 | 0.66 | 1.13 | -1.07 | 0.29 |
| ENSG00000231789 | 0.73 | 0.54 | 0.98 | -2.11 | 0.035 |
| ENSG00000267348 | 0.69 | 0.52 | 0.92 | -2.5 | 0.012 |
| ENSG00000218357 | 0.99 | 0.78 | 1.26 | -0.07 | 0.94 |
| ENSG00000215866 | 1.16 | 0.94 | 1.44 | 1.4 | 0.16 |
| ENSG00000271420 | 0.88 | 0.67 | 1.16 | -0.9 | 0.37 |
| ENSG00000267287 | 0.75 | 0.49 | 1.14 | -1.37 | 0.17 |
| ENSG00000228142 | 0.96 | 0.73 | 1.28 | -0.25 | 0.8 |
| ENSG00000165511 | 1.18 | 0.96 | 1.44 | 1.6 | 0.11 |
| ENSG00000260026 | 0.87 | 0.61 | 1.24 | -0.75 | 0.45 |
| ENSG00000223343 | 0.85 | 0.64 | 1.13 | -1.12 | 0.26 |
| ENSG00000236548 | 1.12 | 0.86 | 1.45 | 0.81 | 0.42 |
| ENSG00000266010 | 1.09 | 0.89 | 1.34 | 0.86 | 0.39 |
| ENSG00000280088 | 0.86 | 0.68 | 1.1 | -1.18 | 0.24 |
| ENSG00000269887 | 0.95 | 0.72 | 1.24 | -0.39 | 0.7 |
| ENSG00000231291 | 1.11 | 0.87 | 1.42 | 0.83 | 0.4 |
| ENSG00000224397 | 1 | 0.79 | 1.26 | -0.01 | 0.99 |
| ENSG00000218416 | 0.85 | 0.64 | 1.12 | -1.17 | 0.24 |
| ENSG00000280327 | 0.77 | 0.56 | 1.05 | -1.63 | 0.1 |
| ENSG00000267277 | 0.86 | 0.64 | 1.14 | -1.05 | 0.3 |
| ENSG00000224739 | 0.89 | 0.7 | 1.15 | -0.89 | 0.38 |
| ENSG00000214922 | 0.93 | 0.71 | 1.2 | -0.59 | 0.56 |
| ENSG00000258082 | 0.93 | 0.72 | 1.2 | -0.58 | 0.56 |
| ENSG00000278385 | 0.83 | 0.64 | 1.07 | -1.44 | 0.15 |
| ENSG00000182165 | 0.79 | 0.61 | 1.01 | -1.91 | 0.056 |
| ENSG00000260577 | 1.03 | 0.81 | 1.31 | 0.25 | 0.81 |
| ENSG00000187185 | 1.11 | 0.9 | 1.37 | 0.97 | 0.33 |
| ENSG00000230454 | 0.69 | 0.51 | 0.93 | -2.45 | 0.014 |
| ENSG00000279427 | 0.91 | 0.7 | 1.17 | -0.75 | 0.45 |
| ENSG00000251169 | 0.89 | 0.69 | 1.15 | -0.87 | 0.38 |
| ENSG00000257808 | 0.98 | 0.74 | 1.3 | -0.12 | 0.9 |
| ENSG00000279227 | 1.25 | 1.01 | 1.55 | 2.03 | 0.042 |
| ENSG00000232638 | 0.8 | 0.61 | 1.05 | -1.61 | 0.11 |
| ENSG00000248846 | 0.97 | 0.78 | 1.23 | -0.22 | 0.83 |
| ENSG00000280916 | 1.11 | 0.87 | 1.4 | 0.83 | 0.41 |
| ENSG00000263280 | 1.04 | 0.81 | 1.35 | 0.33 | 0.74 |
| ENSG00000228544 | 1.12 | 0.87 | 1.46 | 0.88 | 0.38 |
| ENSG00000246228 | 0.93 | 0.73 | 1.19 | -0.55 | 0.58 |
| ENSG00000260136 | 0.83 | 0.63 | 1.09 | -1.33 | 0.18 |
| ENSG00000224853 | 1.17 | 0.92 | 1.48 | 1.26 | 0.21 |
| ENSG00000259704 | 0.87 | 0.67 | 1.14 | -1.02 | 0.31 |
| ENSG00000249599 | 0.97 | 0.76 | 1.24 | -0.25 | 0.8 |
| ENSG00000281162 | 1.09 | 0.88 | 1.33 | 0.78 | 0.44 |
| ENSG00000242516 | 0.97 | 0.74 | 1.26 | -0.23 | 0.82 |
| ENSG00000270419 | 0.77 | 0.57 | 1.05 | -1.67 | 0.095 |
| ENSG00000232739 | 0.96 | 0.74 | 1.24 | -0.32 | 0.75 |
| ENSG00000239445 | 1 | 0.77 | 1.3 | -0.03 | 0.97 |
| ENSG00000225096 | 0.81 | 0.61 | 1.07 | -1.5 | 0.13 |
| ENSG00000235888 | 0.92 | 0.71 | 1.17 | -0.7 | 0.48 |
| ENSG00000278834 | 0.73 | 0.56 | 0.97 | -2.17 | 0.03 |
| ENSG00000257261 | 1.04 | 0.81 | 1.32 | 0.29 | 0.77 |
| ENSG00000260009 | 0.82 | 0.6 | 1.1 | -1.32 | 0.19 |
| ENSG00000254615 | 1.01 | 0.78 | 1.31 | 0.09 | 0.93 |
| ENSG00000280032 | 1.14 | 0.91 | 1.41 | 1.15 | 0.25 |
| ENSG00000267009 | 0.76 | 0.58 | 1.01 | -1.92 | 0.055 |
| ENSG00000120664 | 0.73 | 0.53 | 1.02 | -1.87 | 0.062 |
| ENSG00000231609 | 0.78 | 0.56 | 1.09 | -1.45 | 0.15 |
| ENSG00000234160 | 0.93 | 0.74 | 1.18 | -0.58 | 0.56 |
| ENSG00000261488 | 0.76 | 0.58 | 1 | -1.93 | 0.053 |
| ENSG00000232028 | 0.78 | 0.57 | 1.07 | -1.52 | 0.13 |
| ENSG00000259725 | 0.82 | 0.62 | 1.08 | -1.41 | 0.16 |
| ENSG00000259459 | 0.78 | 0.61 | 1 | -1.95 | 0.052 |
| ENSG00000225177 | 1.08 | 0.83 | 1.39 | 0.56 | 0.57 |
| ENSG00000272620 | 0.83 | 0.61 | 1.12 | -1.21 | 0.23 |
| ENSG00000164385 | 0.94 | 0.71 | 1.25 | -0.4 | 0.69 |
| ENSG00000254290 | 0.83 | 0.63 | 1.09 | -1.35 | 0.18 |
| ENSG00000235049 | 0.91 | 0.69 | 1.2 | -0.68 | 0.5 |
| ENSG00000243176 | 0.82 | 0.62 | 1.09 | -1.38 | 0.17 |
| ENSG00000248538 | 1.02 | 0.81 | 1.27 | 0.15 | 0.88 |
| ENSG00000223799 | 0.96 | 0.73 | 1.26 | -0.27 | 0.79 |
| ENSG00000254006 | 1.27 | 0.99 | 1.62 | 1.88 | 0.06 |
| ENSG00000241679 | 0.99 | 0.8 | 1.23 | -0.1 | 0.92 |
| ENSG00000227036 | 1.18 | 0.92 | 1.52 | 1.28 | 0.2 |
| ENSG00000272405 | 0.99 | 0.77 | 1.28 | -0.07 | 0.95 |
| ENSG00000225764 | 0.73 | 0.49 | 1.1 | -1.51 | 0.13 |
| ENSG00000260267 | 0.85 | 0.65 | 1.12 | -1.15 | 0.25 |
| ENSG00000235078 | 0.89 | 0.65 | 1.22 | -0.7 | 0.48 |
| ENSG00000268615 | 0.92 | 0.71 | 1.19 | -0.63 | 0.53 |
| ENSG00000236751 | 0.88 | 0.63 | 1.21 | -0.8 | 0.42 |
| ENSG00000246560 | 1.02 | 0.79 | 1.31 | 0.15 | 0.88 |
| ENSG00000226835 | 0.84 | 0.61 | 1.15 | -1.08 | 0.28 |
| ENSG00000279822 | 1.04 | 0.88 | 1.22 | 0.41 | 0.68 |
| ENSG00000271576 | 0.88 | 0.66 | 1.19 | -0.83 | 0.41 |
| ENSG00000232352 | 0.61 | 0.45 | 0.82 | -3.23 | 0.0012 |
| ENSG00000226101 | 0.85 | 0.61 | 1.19 | -0.95 | 0.34 |
| ENSG00000224842 | 0.83 | 0.63 | 1.09 | -1.35 | 0.18 |
| ENSG00000253959 | 0.77 | 0.59 | 1.01 | -1.87 | 0.062 |
| ENSG00000223808 | 0.96 | 0.76 | 1.23 | -0.29 | 0.77 |
| ENSG00000270362 | 0.97 | 0.75 | 1.25 | -0.26 | 0.79 |
| ENSG00000232803 | 1.03 | 0.78 | 1.37 | 0.22 | 0.83 |
| ENSG00000231439 | 1.02 | 0.8 | 1.32 | 0.19 | 0.85 |
| ENSG00000248399 | 0.69 | 0.51 | 0.94 | -2.37 | 0.018 |
| ENSG00000234918 | 0.9 | 0.71 | 1.13 | -0.93 | 0.35 |
| ENSG00000240057 | 0.95 | 0.7 | 1.29 | -0.34 | 0.73 |
| ENSG00000254416 | 1.07 | 0.77 | 1.48 | 0.39 | 0.7 |
| ENSG00000235545 | 0.89 | 0.68 | 1.17 | -0.82 | 0.41 |
| ENSG00000233823 | 0.97 | 0.75 | 1.26 | -0.21 | 0.83 |
| ENSG00000167912 | 0.87 | 0.64 | 1.17 | -0.94 | 0.35 |
| ENSG00000204446 | 1.02 | 0.81 | 1.3 | 0.19 | 0.85 |
| ENSG00000255133 | 1.15 | 0.93 | 1.43 | 1.27 | 0.21 |
| ENSG00000226816 | 1.06 | 0.81 | 1.38 | 0.41 | 0.68 |
| ENSG00000203711 | 1.04 | 0.78 | 1.38 | 0.26 | 0.79 |
| ENSG00000254027 | 1.14 | 0.9 | 1.43 | 1.07 | 0.29 |
| ENSG00000225361 | 1.02 | 0.8 | 1.31 | 0.15 | 0.88 |
| ENSG00000276850 | 0.93 | 0.71 | 1.24 | -0.47 | 0.63 |
| ENSG00000280693 | 1.03 | 0.81 | 1.31 | 0.25 | 0.8 |
| ENSG00000224424 | 0.94 | 0.72 | 1.21 | -0.51 | 0.61 |
| ENSG00000223979 | 0.96 | 0.78 | 1.18 | -0.39 | 0.7 |
| ENSG00000229124 | 0.8 | 0.61 | 1.06 | -1.58 | 0.11 |
| ENSG00000257696 | 1.06 | 0.83 | 1.35 | 0.47 | 0.64 |
| ENSG00000251141 | 0.8 | 0.61 | 1.04 | -1.69 | 0.091 |
| ENSG00000257135 | 0.88 | 0.66 | 1.16 | -0.89 | 0.37 |
| ENSG00000236404 | 0.78 | 0.54 | 1.11 | -1.38 | 0.17 |
| ENSG00000267079 | 0.72 | 0.48 | 1.09 | -1.54 | 0.12 |
| ENSG00000234869 | 0.78 | 0.59 | 1.03 | -1.73 | 0.084 |
| ENSG00000267077 | 0.75 | 0.54 | 1.04 | -1.71 | 0.087 |
| ENSG00000236882 | 1.13 | 0.93 | 1.38 | 1.21 | 0.23 |
| ENSG00000235072 | 0.91 | 0.67 | 1.22 | -0.65 | 0.51 |
| ENSG00000262468 | 0.8 | 0.61 | 1.07 | -1.51 | 0.13 |
| ENSG00000280099 | 0.96 | 0.72 | 1.26 | -0.32 | 0.75 |
| ENSG00000280187 | 0.87 | 0.66 | 1.13 | -1.06 | 0.29 |
| ENSG00000254035 | 0.97 | 0.75 | 1.25 | -0.23 | 0.82 |
| ENSG00000254602 | 0.82 | 0.58 | 1.14 | -1.19 | 0.24 |
| ENSG00000278621 | 0.86 | 0.67 | 1.1 | -1.23 | 0.22 |
| ENSG00000250244 | 0.98 | 0.74 | 1.28 | -0.18 | 0.86 |
| ENSG00000229891 | 0.88 | 0.68 | 1.15 | -0.92 | 0.36 |
| ENSG00000233621 | 0.92 | 0.71 | 1.19 | -0.65 | 0.52 |
| ENSG00000233006 | 0.79 | 0.6 | 1.05 | -1.6 | 0.11 |
| ENSG00000255857 | 0.86 | 0.66 | 1.12 | -1.13 | 0.26 |
| ENSG00000239311 | 1.07 | 0.86 | 1.33 | 0.61 | 0.54 |
| ENSG00000231817 | 0.86 | 0.58 | 1.3 | -0.7 | 0.48 |
| ENSG00000257829 | 1.13 | 0.91 | 1.4 | 1.1 | 0.27 |
| ENSG00000237523 | 1.11 | 0.87 | 1.41 | 0.82 | 0.41 |
| ENSG00000275186 | 0.67 | 0.4 | 1.1 | -1.58 | 0.11 |
| ENSG00000257718 | 0.99 | 0.73 | 1.33 | -0.08 | 0.94 |
| ENSG00000204049 | 0.86 | 0.66 | 1.13 | -1.07 | 0.28 |
| ENSG00000267052 | 0.59 | 0.37 | 0.94 | -2.22 | 0.026 |
| ENSG00000253125 | 0.78 | 0.6 | 1.02 | -1.85 | 0.065 |
| ENSG00000241359 | 0.75 | 0.58 | 0.98 | -2.07 | 0.038 |
| ENSG00000230978 | 0.8 | 0.6 | 1.05 | -1.6 | 0.11 |
| ENSG00000275874 | 1.08 | 0.82 | 1.43 | 0.55 | 0.58 |
| ENSG00000272068 | 0.94 | 0.72 | 1.22 | -0.48 | 0.63 |
| ENSG00000179066 | 1.06 | 0.83 | 1.36 | 0.5 | 0.62 |
| ENSG00000277182 | 0.79 | 0.57 | 1.1 | -1.39 | 0.17 |
| ENSG00000225431 | 1.15 | 0.93 | 1.43 | 1.32 | 0.19 |
| ENSG00000261183 | 0.81 | 0.62 | 1.04 | -1.63 | 0.1 |
| ENSG00000261801 | 0.99 | 0.77 | 1.27 | -0.07 | 0.94 |
| ENSG00000250643 | 1.06 | 0.83 | 1.35 | 0.49 | 0.63 |
| ENSG00000163364 | 0.89 | 0.7 | 1.13 | -0.96 | 0.33 |
| ENSG00000273062 | 0.84 | 0.66 | 1.08 | -1.34 | 0.18 |
| ENSG00000279095 | 0.95 | 0.73 | 1.25 | -0.34 | 0.73 |
| ENSG00000271727 | 0.6 | 0.4 | 0.9 | -2.47 | 0.013 |
| ENSG00000235584 | 0.93 | 0.73 | 1.19 | -0.56 | 0.57 |
| ENSG00000268496 | 1 | 0.78 | 1.28 | 0.01 | 0.99 |
| ENSG00000268049 | 0.7 | 0.53 | 0.92 | -2.54 | 0.011 |
| ENSG00000259793 | 0.91 | 0.71 | 1.17 | -0.76 | 0.44 |
| ENSG00000258910 | 1.13 | 0.91 | 1.41 | 1.1 | 0.27 |
| ENSG00000253509 | 1.09 | 0.84 | 1.42 | 0.63 | 0.53 |
| ENSG00000266846 | 0.79 | 0.56 | 1.1 | -1.4 | 0.16 |
| ENSG00000244265 | 0.67 | 0.49 | 0.91 | -2.54 | 0.011 |
| ENSG00000185904 | 1.14 | 0.91 | 1.42 | 1.15 | 0.25 |
| ENSG00000260228 | 1.15 | 0.9 | 1.45 | 1.13 | 0.26 |
| ENSG00000279778 | 0.88 | 0.64 | 1.2 | -0.8 | 0.42 |
| ENSG00000232767 | 0.94 | 0.73 | 1.2 | -0.51 | 0.61 |
| ENSG00000231889 | 0.86 | 0.64 | 1.15 | -1.05 | 0.3 |
| ENSG00000254973 | 1.13 | 0.89 | 1.45 | 1.01 | 0.31 |
| ENSG00000272518 | 0.98 | 0.75 | 1.29 | -0.13 | 0.89 |
| ENSG00000267288 | 0.81 | 0.6 | 1.08 | -1.42 | 0.16 |
| ENSG00000248932 | 0.87 | 0.67 | 1.12 | -1.09 | 0.27 |
| ENSG00000234233 | 0.85 | 0.66 | 1.11 | -1.2 | 0.23 |
| ENSG00000259430 | 0.71 | 0.46 | 1.08 | -1.6 | 0.11 |
| ENSG00000258479 | 0.87 | 0.62 | 1.24 | -0.75 | 0.45 |
| ENSG00000261534 | 1.05 | 0.81 | 1.35 | 0.36 | 0.72 |
| ENSG00000271755 | 0.86 | 0.64 | 1.16 | -0.97 | 0.33 |
| ENSG00000246898 | 1.03 | 0.82 | 1.3 | 0.26 | 0.79 |
| ENSG00000260578 | 1.1 | 0.95 | 1.29 | 1.27 | 0.2 |
| ENSG00000245857 | 0.9 | 0.64 | 1.25 | -0.64 | 0.52 |
| ENSG00000178734 | 0.79 | 0.56 | 1.11 | -1.37 | 0.17 |
| ENSG00000248994 | 0.77 | 0.56 | 1.07 | -1.54 | 0.12 |
| ENSG00000267603 | 0.78 | 0.44 | 1.39 | -0.85 | 0.4 |
| ENSG00000274818 | 0.79 | 0.6 | 1.05 | -1.6 | 0.11 |
| ENSG00000261390 | 0.83 | 0.61 | 1.12 | -1.23 | 0.22 |
| ENSG00000272501 | 0.82 | 0.62 | 1.08 | -1.44 | 0.15 |
| ENSG00000236213 | 0.59 | 0.41 | 0.87 | -2.66 | 0.0078 |
| ENSG00000223534 | 0.67 | 0.51 | 0.88 | -2.84 | 0.0045 |
| ENSG00000231682 | 0.95 | 0.72 | 1.24 | -0.37 | 0.71 |
| ENSG00000254135 | 1 | 0.79 | 1.26 | 0.01 | 0.99 |
| ENSG00000229498 | 0.94 | 0.69 | 1.27 | -0.4 | 0.69 |
| ENSG00000236961 | 0.95 | 0.67 | 1.35 | -0.28 | 0.78 |
| ENSG00000260244 | 1.04 | 0.82 | 1.33 | 0.34 | 0.73 |
| ENSG00000254675 | 0.96 | 0.72 | 1.27 | -0.31 | 0.75 |
| ENSG00000242370 | 0.88 | 0.67 | 1.16 | -0.9 | 0.37 |
| ENSG00000229224 | 0.47 | 0.25 | 0.9 | -2.27 | 0.023 |
| ENSG00000273124 | 0.75 | 0.52 | 1.09 | -1.51 | 0.13 |
| ENSG00000250132 | 0.92 | 0.7 | 1.22 | -0.56 | 0.58 |
| ENSG00000259446 | 1.27 | 0.97 | 1.66 | 1.72 | 0.086 |
| ENSG00000237975 | 1.03 | 0.8 | 1.32 | 0.22 | 0.83 |
| ENSG00000243953 | 0.72 | 0.43 | 1.18 | -1.31 | 0.19 |
| ENSG00000234810 | 0.81 | 0.51 | 1.27 | -0.93 | 0.35 |
| ENSG00000233332 | 1.19 | 0.96 | 1.48 | 1.59 | 0.11 |
| ENSG00000272505 | 0.7 | 0.49 | 1.02 | -1.88 | 0.06 |
| ENSG00000277135 | 0.65 | 0.44 | 0.95 | -2.25 | 0.024 |
| ENSG00000251576 | 0.74 | 0.52 | 1.07 | -1.61 | 0.11 |
| ENSG00000279571 | 0.85 | 0.64 | 1.13 | -1.11 | 0.27 |
| ENSG00000255021 | 0.63 | 0.41 | 0.97 | -2.1 | 0.036 |
| ENSG00000259363 | 1 | 0.78 | 1.28 | -0.03 | 0.98 |
| ENSG00000229694 | 0.95 | 0.72 | 1.26 | -0.34 | 0.74 |
| ENSG00000233574 | 0.89 | 0.67 | 1.19 | -0.77 | 0.44 |
| ENSG00000250266 | 0.53 | 0.2 | 1.42 | -1.26 | 0.21 |
| ENSG00000229647 | 0.95 | 0.73 | 1.25 | -0.34 | 0.73 |
| ENSG00000272316 | 0.73 | 0.55 | 0.97 | -2.14 | 0.032 |
| ENSG00000232063 | 1.09 | 0.86 | 1.38 | 0.71 | 0.48 |
| ENSG00000260633 | 0.81 | 0.6 | 1.09 | -1.41 | 0.16 |
| ENSG00000250692 | 1 | 0.8 | 1.25 | -0.01 | 0.99 |
| ENSG00000151303 | 1.01 | 0.8 | 1.29 | 0.1 | 0.92 |
| ENSG00000236036 | 1.22 | 0.98 | 1.51 | 1.8 | 0.072 |
| ENSG00000233554 | 0.82 | 0.62 | 1.07 | -1.44 | 0.15 |
| ENSG00000235434 | 0.77 | 0.52 | 1.14 | -1.32 | 0.19 |
| ENSG00000227544 | 0.78 | 0.58 | 1.04 | -1.69 | 0.091 |
| ENSG00000231170 | 0.87 | 0.67 | 1.13 | -1.05 | 0.29 |
| ENSG00000272416 | 0.8 | 0.61 | 1.05 | -1.6 | 0.11 |
| ENSG00000245105 | 0.94 | 0.75 | 1.19 | -0.52 | 0.6 |
| ENSG00000275569 | 0.81 | 0.59 | 1.1 | -1.37 | 0.17 |
| ENSG00000267583 | 1.04 | 0.8 | 1.35 | 0.29 | 0.77 |
| ENSG00000236915 | 0.74 | 0.54 | 1.01 | -1.87 | 0.061 |
| ENSG00000251399 | 1.08 | 0.95 | 1.25 | 1.16 | 0.25 |
| ENSG00000234577 | 0.89 | 0.68 | 1.17 | -0.81 | 0.42 |
| ENSG00000257551 | 0.99 | 0.76 | 1.29 | -0.08 | 0.94 |
| ENSG00000281128 | 0.93 | 0.69 | 1.27 | -0.45 | 0.65 |
| ENSG00000237187 | 1.21 | 0.94 | 1.56 | 1.45 | 0.15 |
| ENSG00000254041 | 0.91 | 0.69 | 1.2 | -0.66 | 0.51 |
| ENSG00000255920 | 0.86 | 0.64 | 1.15 | -1.03 | 0.3 |
| ENSG00000258498 | 0.65 | 0.42 | 0.98 | -2.03 | 0.042 |
| ENSG00000253608 | 1.2 | 0.9 | 1.59 | 1.25 | 0.21 |
| ENSG00000226530 | 0.9 | 0.68 | 1.17 | -0.8 | 0.42 |
| ENSG00000238198 | 0.69 | 0.48 | 1 | -1.95 | 0.052 |
| ENSG00000223458 | 0.91 | 0.67 | 1.24 | -0.6 | 0.55 |
| ENSG00000256654 | 0.57 | 0.31 | 1.05 | -1.82 | 0.069 |
| ENSG00000280241 | 1.34 | 1.08 | 1.67 | 2.6 | 0.0093 |
| ENSG00000272797 | 0.68 | 0.43 | 1.06 | -1.72 | 0.085 |
| ENSG00000260917 | 0.93 | 0.71 | 1.22 | -0.5 | 0.62 |
| ENSG00000258819 | 0.84 | 0.63 | 1.13 | -1.15 | 0.25 |
| ENSG00000254810 | 1.01 | 0.79 | 1.29 | 0.08 | 0.94 |
| ENSG00000254389 | 1.19 | 0.93 | 1.51 | 1.41 | 0.16 |
| ENSG00000248896 | 0.77 | 0.58 | 1.02 | -1.79 | 0.073 |
| ENSG00000279444 | 0.97 | 0.72 | 1.32 | -0.2 | 0.84 |
| ENSG00000258636 | 0.85 | 0.64 | 1.12 | -1.14 | 0.25 |
| ENSG00000279499 | 0.94 | 0.73 | 1.22 | -0.44 | 0.66 |
| ENSG00000267519 | 0.87 | 0.67 | 1.13 | -1.05 | 0.3 |
| ENSG00000279526 | 0.85 | 0.6 | 1.2 | -0.93 | 0.35 |
| ENSG00000279026 | 0.95 | 0.72 | 1.24 | -0.39 | 0.7 |
| ENSG00000253688 | 1.01 | 0.8 | 1.29 | 0.11 | 0.91 |
| ENSG00000273409 | 0.94 | 0.69 | 1.26 | -0.42 | 0.67 |
| ENSG00000257167 | 0.99 | 0.8 | 1.23 | -0.07 | 0.95 |
| ENSG00000273162 | 1.07 | 0.84 | 1.37 | 0.58 | 0.56 |
| ENSG00000265415 | 1.31 | 1.03 | 1.68 | 2.18 | 0.029 |
| ENSG00000226622 | 1.03 | 0.75 | 1.41 | 0.15 | 0.88 |
| ENSG00000187951 | 0.91 | 0.71 | 1.17 | -0.72 | 0.47 |
| ENSG00000249752 | 0.89 | 0.66 | 1.2 | -0.75 | 0.45 |
| ENSG00000233117 | 1.24 | 0.97 | 1.58 | 1.75 | 0.079 |
| ENSG00000258441 | 0.77 | 0.58 | 1.03 | -1.76 | 0.078 |
| ENSG00000259869 | 1.3 | 1.06 | 1.6 | 2.55 | 0.011 |
| ENSG00000259732 | 0.88 | 0.65 | 1.19 | -0.84 | 0.4 |
| ENSG00000277144 | 0.83 | 0.61 | 1.13 | -1.19 | 0.23 |
| ENSG00000227220 | 0.79 | 0.55 | 1.14 | -1.26 | 0.21 |
| ENSG00000227959 | 1.04 | 0.83 | 1.32 | 0.37 | 0.71 |
| ENSG00000227475 | 0.95 | 0.71 | 1.27 | -0.33 | 0.74 |
| ENSG00000272808 | 0.95 | 0.71 | 1.27 | -0.32 | 0.75 |
| ENSG00000272870 | 0.92 | 0.7 | 1.21 | -0.59 | 0.55 |
| ENSG00000278456 | 1.02 | 0.76 | 1.35 | 0.12 | 0.91 |
| ENSG00000166770 | 0.87 | 0.69 | 1.1 | -1.17 | 0.24 |
| ENSG00000279821 | 0.62 | 0.38 | 1.01 | -1.93 | 0.054 |
| ENSG00000272537 | 0.92 | 0.71 | 1.2 | -0.59 | 0.56 |
| ENSG00000274565 | 0.9 | 0.66 | 1.23 | -0.66 | 0.51 |
| ENSG00000280719 | 1.04 | 0.79 | 1.37 | 0.29 | 0.77 |
| ENSG00000256894 | 0.98 | 0.78 | 1.23 | -0.16 | 0.88 |
| ENSG00000254369 | 0.88 | 0.64 | 1.22 | -0.76 | 0.45 |
| ENSG00000270504 | 1.11 | 0.86 | 1.43 | 0.78 | 0.44 |
| ENSG00000273669 | 0.81 | 0.62 | 1.06 | -1.53 | 0.13 |
| ENSG00000235840 | 0.25 | 0.1 | 0.62 | -2.98 | 0.0029 |
| ENSG00000273442 | 0.9 | 0.7 | 1.16 | -0.78 | 0.43 |
| ENSG00000250921 | 0.97 | 0.83 | 1.13 | -0.4 | 0.69 |
| ENSG00000272734 | 0.9 | 0.69 | 1.17 | -0.81 | 0.42 |
| ENSG00000224609 | 1.07 | 0.93 | 1.23 | 1 | 0.32 |
| ENSG00000254943 | 0.01 | 0 | Inf | 0 | 1 |
| ENSG00000240666 | 0.87 | 0.65 | 1.16 | -0.98 | 0.33 |
| ENSG00000234336 | 0.78 | 0.56 | 1.08 | -1.48 | 0.14 |
| ENSG00000271614 | 0.79 | 0.59 | 1.05 | -1.63 | 0.1 |
| ENSG00000278989 | 0.68 | 0.49 | 0.92 | -2.47 | 0.013 |
| ENSG00000221571 | 0.73 | 0.52 | 1.04 | -1.74 | 0.081 |
| ENSG00000234688 | 1.17 | 0.86 | 1.61 | 1 | 0.32 |
| ENSG00000228139 | 0.99 | 0.77 | 1.26 | -0.12 | 0.91 |
| ENSG00000226542 | 0.85 | 0.62 | 1.17 | -1 | 0.32 |
| ENSG00000242611 | 1.04 | 0.8 | 1.35 | 0.28 | 0.78 |
| ENSG00000277199 | 0.81 | 0.52 | 1.25 | -0.97 | 0.33 |
| ENSG00000233766 | 0.83 | 0.61 | 1.13 | -1.18 | 0.24 |
| ENSG00000272123 | 0.75 | 0.57 | 0.99 | -2.06 | 0.039 |
| ENSG00000183154 | 1.01 | 0.78 | 1.31 | 0.1 | 0.92 |
| ENSG00000267543 | 0.85 | 0.66 | 1.11 | -1.18 | 0.24 |
| ENSG00000231160 | 0.75 | 0.56 | 1 | -1.93 | 0.053 |
| ENSG00000203392 | 0.99 | 0.74 | 1.32 | -0.09 | 0.93 |
| ENSG00000251189 | 0.87 | 0.64 | 1.18 | -0.89 | 0.37 |
| ENSG00000234661 | 1.08 | 0.91 | 1.29 | 0.87 | 0.38 |
| ENSG00000260329 | 0.84 | 0.65 | 1.08 | -1.34 | 0.18 |
| ENSG00000250282 | 1.11 | 0.92 | 1.34 | 1.09 | 0.27 |
| ENSG00000272650 | 0.92 | 0.71 | 1.19 | -0.63 | 0.53 |
| ENSG00000253647 | 0.7 | 0.45 | 1.1 | -1.55 | 0.12 |
| ENSG00000263753 | 0.73 | 0.58 | 0.93 | -2.58 | 0.0098 |
| ENSG00000226009 | 0.96 | 0.74 | 1.25 | -0.29 | 0.77 |
| ENSG00000248275 | 0.82 | 0.63 | 1.07 | -1.45 | 0.15 |
| ENSG00000224032 | 0.81 | 0.63 | 1.03 | -1.75 | 0.08 |
| ENSG00000260192 | 0.93 | 0.71 | 1.21 | -0.57 | 0.57 |
| ENSG00000272801 | 0.95 | 0.72 | 1.26 | -0.33 | 0.74 |
| ENSG00000237612 | 0.73 | 0.46 | 1.17 | -1.31 | 0.19 |
| ENSG00000272631 | 0.96 | 0.75 | 1.25 | -0.27 | 0.79 |
| ENSG00000228063 | 0.85 | 0.65 | 1.12 | -1.14 | 0.25 |
| ENSG00000228035 | 0.68 | 0.29 | 1.59 | -0.89 | 0.38 |
| ENSG00000272508 | 0.94 | 0.72 | 1.23 | -0.42 | 0.68 |
| ENSG00000237159 | 1.12 | 0.88 | 1.42 | 0.94 | 0.35 |
| ENSG00000236859 | 0.7 | 0.55 | 0.9 | -2.78 | 0.0054 |
| ENSG00000270953 | 0.72 | 0.53 | 0.98 | -2.11 | 0.035 |
| ENSG00000226849 | 0.74 | 0.54 | 1.01 | -1.88 | 0.06 |
| ENSG00000232930 | 0.66 | 0.38 | 1.12 | -1.54 | 0.12 |
| ENSG00000268287 | 0.7 | 0.45 | 1.08 | -1.61 | 0.11 |
| ENSG00000270959 | 0.84 | 0.64 | 1.09 | -1.29 | 0.2 |
| ENSG00000224568 | 0.73 | 0.2 | 2.63 | -0.48 | 0.63 |
| ENSG00000232328 | 1.17 | 0.97 | 1.43 | 1.61 | 0.11 |
| ENSG00000280739 | 0.85 | 0.66 | 1.09 | -1.29 | 0.2 |
| ENSG00000250572 | 1 | 0.76 | 1.31 | 0 | 1 |
| ENSG00000262898 | 1.06 | 0.81 | 1.39 | 0.42 | 0.68 |
| ENSG00000250685 | 0.78 | 0.52 | 1.17 | -1.2 | 0.23 |
| ENSG00000267339 | 0.93 | 0.65 | 1.33 | -0.4 | 0.69 |
| ENSG00000230838 | 1.26 | 0.98 | 1.61 | 1.8 | 0.073 |
| ENSG00000259285 | 0.85 | 0.62 | 1.15 | -1.06 | 0.29 |
| ENSG00000234884 | 0.68 | 0.48 | 0.96 | -2.17 | 0.03 |
| ENSG00000247572 | 0.8 | 0.62 | 1.04 | -1.69 | 0.09 |
| ENSG00000261707 | 0.95 | 0.73 | 1.23 | -0.41 | 0.68 |
| ENSG00000244738 | 0.71 | 0.42 | 1.19 | -1.3 | 0.19 |
| ENSG00000230937 | 0.73 | 0.57 | 0.95 | -2.38 | 0.017 |
| ENSG00000279622 | 0.79 | 0.56 | 1.13 | -1.3 | 0.19 |
| ENSG00000272549 | 0.77 | 0.49 | 1.23 | -1.09 | 0.28 |
| ENSG00000249436 | 0.91 | 0.62 | 1.33 | -0.49 | 0.62 |
| ENSG00000230790 | 1.01 | 0.77 | 1.33 | 0.09 | 0.93 |
| ENSG00000250303 | 0.91 | 0.7 | 1.19 | -0.67 | 0.51 |
| ENSG00000224613 | 0.97 | 0.66 | 1.42 | -0.17 | 0.86 |
| ENSG00000230735 | 1.06 | 0.83 | 1.34 | 0.45 | 0.65 |
| ENSG00000251396 | 1.16 | 0.92 | 1.47 | 1.24 | 0.22 |
| ENSG00000234754 | 0.89 | 0.57 | 1.39 | -0.52 | 0.6 |
| ENSG00000273403 | 0.9 | 0.68 | 1.19 | -0.75 | 0.45 |
| ENSG00000230928 | 0.88 | 0.58 | 1.32 | -0.63 | 0.53 |
| ENSG00000267038 | 1.1 | 0.87 | 1.38 | 0.81 | 0.42 |
| ENSG00000232814 | 0.71 | 0.5 | 1 | -1.96 | 0.051 |
| ENSG00000257084 | 0.69 | 0.52 | 0.92 | -2.5 | 0.012 |
| ENSG00000279803 | 1.14 | 0.91 | 1.42 | 1.14 | 0.25 |
| ENSG00000277089 | 0.89 | 0.69 | 1.16 | -0.86 | 0.39 |
| ENSG00000237352 | 0.74 | 0.52 | 1.06 | -1.63 | 0.1 |
| ENSG00000196167 | 1.05 | 0.84 | 1.32 | 0.46 | 0.64 |
| ENSG00000261490 | 0.96 | 0.7 | 1.3 | -0.27 | 0.78 |
| ENSG00000234235 | 0.89 | 0.63 | 1.27 | -0.64 | 0.52 |
| ENSG00000234292 | 1.11 | 0.88 | 1.4 | 0.9 | 0.37 |
| ENSG00000273821 | 0.9 | 0.67 | 1.19 | -0.76 | 0.45 |
| ENSG00000271584 | 0.98 | 0.77 | 1.24 | -0.18 | 0.86 |
| ENSG00000248362 | 0.88 | 0.67 | 1.15 | -0.92 | 0.36 |
| ENSG00000251320 | 0.87 | 0.66 | 1.15 | -0.99 | 0.32 |
| ENSG00000261706 | 1.25 | 1.01 | 1.55 | 2.02 | 0.043 |
| ENSG00000230333 | 1.06 | 0.9 | 1.25 | 0.68 | 0.5 |
| ENSG00000265554 | 1.11 | 0.74 | 1.66 | 0.5 | 0.62 |
| ENSG00000223626 | 0.55 | 0.1 | 3 | -0.69 | 0.49 |
| ENSG00000250240 | 0.94 | 0.72 | 1.21 | -0.5 | 0.62 |
| ENSG00000273923 | 1.05 | 0.84 | 1.32 | 0.42 | 0.67 |
| ENSG00000231013 | 1.27 | 0.95 | 1.69 | 1.64 | 0.1 |
| ENSG00000250968 | 0 | 0 | Inf | 0 | 1 |
| ENSG00000272855 | 1.3 | 1.04 | 1.62 | 2.35 | 0.019 |
| ENSG00000272909 | 0.95 | 0.73 | 1.23 | -0.4 | 0.69 |
| ENSG00000255455 | 0.96 | 0.75 | 1.24 | -0.31 | 0.75 |
| ENSG00000260442 | 0.93 | 0.7 | 1.23 | -0.51 | 0.61 |
| ENSG00000272172 | 0.92 | 0.7 | 1.21 | -0.62 | 0.53 |
| ENSG00000231948 | 1.19 | 0.94 | 1.51 | 1.44 | 0.15 |
| ENSG00000253661 | 0.99 | 0.76 | 1.28 | -0.08 | 0.94 |
| ENSG00000233817 | 1.1 | 0.85 | 1.42 | 0.73 | 0.47 |
| ENSG00000233858 | 0.79 | 0.5 | 1.26 | -0.98 | 0.33 |
| ENSG00000254366 | 0.91 | 0.61 | 1.34 | -0.5 | 0.62 |
| ENSG00000249464 | 1.25 | 0.94 | 1.67 | 1.54 | 0.12 |
| ENSG00000235427 | 0.86 | 0.64 | 1.14 | -1.06 | 0.29 |
| ENSG00000224468 | 0.9 | 0.69 | 1.18 | -0.74 | 0.46 |
| ENSG00000249487 | 0.88 | 0.52 | 1.51 | -0.45 | 0.65 |
| ENSG00000261437 | 0.95 | 0.72 | 1.25 | -0.38 | 0.7 |
| ENSG00000255197 | 1.04 | 0.81 | 1.33 | 0.28 | 0.78 |
| ENSG00000235123 | 0.95 | 0.74 | 1.22 | -0.4 | 0.69 |
| ENSG00000261324 | 0.95 | 0.75 | 1.22 | -0.37 | 0.71 |
| ENSG00000224259 | 1.14 | 0.91 | 1.42 | 1.16 | 0.25 |
| ENSG00000232677 | 0.97 | 0.75 | 1.27 | -0.19 | 0.85 |
| ENSG00000227117 | 0.97 | 0.64 | 1.46 | -0.15 | 0.88 |
| ENSG00000250802 | 1.02 | 0.8 | 1.3 | 0.17 | 0.87 |
| ENSG00000273177 | 1.03 | 0.77 | 1.38 | 0.21 | 0.83 |
| ENSG00000259244 | 0.75 | 0.56 | 1.01 | -1.91 | 0.056 |
| ENSG00000224239 | 0.68 | 0.43 | 1.07 | -1.67 | 0.095 |
| ENSG00000224957 | 0.84 | 0.54 | 1.31 | -0.76 | 0.45 |
| ENSG00000259877 | 0.95 | 0.72 | 1.26 | -0.36 | 0.72 |
| ENSG00000248890 | 0.66 | 0.47 | 0.93 | -2.39 | 0.017 |
| ENSG00000259623 | 0.9 | 0.7 | 1.15 | -0.84 | 0.4 |
| ENSG00000250048 | 0.64 | 0.4 | 1.03 | -1.85 | 0.064 |
| ENSG00000270212 | 1.26 | 1.06 | 1.5 | 2.56 | 0.011 |
| ENSG00000227509 | 0.85 | 0.56 | 1.28 | -0.78 | 0.43 |
| ENSG00000243479 | 1.07 | 0.83 | 1.37 | 0.51 | 0.61 |
| ENSG00000231742 | 0.91 | 0.67 | 1.24 | -0.59 | 0.55 |
| ENSG00000272264 | 1.17 | 0.93 | 1.48 | 1.37 | 0.17 |
| ENSG00000279406 | 0.96 | 0.77 | 1.19 | -0.38 | 0.71 |
| ENSG00000260077 | 0.95 | 0.73 | 1.23 | -0.41 | 0.68 |
| ENSG00000227188 | 0.94 | 0.67 | 1.31 | -0.38 | 0.7 |
| ENSG00000274248 | 1.05 | 0.85 | 1.29 | 0.43 | 0.67 |
| ENSG00000237987 | 0.76 | 0.47 | 1.24 | -1.09 | 0.27 |
| ENSG00000242396 | 0.97 | 0.73 | 1.28 | -0.24 | 0.81 |
| ENSG00000249859 | 0.84 | 0.63 | 1.13 | -1.16 | 0.25 |
| ENSG00000232093 | 0.87 | 0.65 | 1.16 | -0.96 | 0.34 |
| ENSG00000271795 | 0.75 | 0.55 | 1.01 | -1.89 | 0.058 |
| ENSG00000280047 | 0.93 | 0.71 | 1.2 | -0.57 | 0.57 |
| ENSG00000235501 | 0.97 | 0.75 | 1.25 | -0.26 | 0.79 |
| ENSG00000263293 | 0.95 | 0.76 | 1.2 | -0.4 | 0.69 |
| ENSG00000251359 | 0.87 | 0.66 | 1.14 | -1.03 | 0.3 |
| ENSG00000267374 | 0.96 | 0.75 | 1.23 | -0.31 | 0.76 |
| ENSG00000245614 | 0.86 | 0.65 | 1.15 | -1.01 | 0.31 |
| ENSG00000260920 | 1.16 | 0.92 | 1.47 | 1.29 | 0.2 |
| ENSG00000261061 | 1.21 | 0.94 | 1.55 | 1.5 | 0.13 |
| ENSG00000271936 | 1.09 | 0.86 | 1.38 | 0.74 | 0.46 |
| ENSG00000276855 | 0.78 | 0.61 | 1 | -1.95 | 0.051 |
| ENSG00000274825 | 1.03 | 0.81 | 1.31 | 0.25 | 0.8 |
| ENSG00000273209 | 0.73 | 0.51 | 1.05 | -1.71 | 0.087 |
| ENSG00000229167 | 1.13 | 0.86 | 1.48 | 0.9 | 0.37 |
| ENSG00000259370 | 1.04 | 0.82 | 1.33 | 0.32 | 0.75 |
| ENSG00000204682 | 1.11 | 0.89 | 1.39 | 0.94 | 0.34 |
| ENSG00000237128 | 1.05 | 0.81 | 1.36 | 0.38 | 0.7 |
| ENSG00000236120 | 1.3 | 1.05 | 1.62 | 2.38 | 0.017 |
| ENSG00000250208 | 0.71 | 0.49 | 1.02 | -1.87 | 0.061 |
| ENSG00000230798 | 0.82 | 0.63 | 1.06 | -1.5 | 0.13 |
| ENSG00000171889 | 0.98 | 0.73 | 1.32 | -0.13 | 0.9 |
| ENSG00000218018 | 0.81 | 0.6 | 1.1 | -1.36 | 0.17 |
| ENSG00000228084 | 0.98 | 0.76 | 1.27 | -0.12 | 0.9 |
| ENSG00000279369 | 0.85 | 0.63 | 1.15 | -1.04 | 0.3 |
| ENSG00000260271 | 0.81 | 0.57 | 1.14 | -1.22 | 0.22 |
| ENSG00000236094 | 0.95 | 0.72 | 1.25 | -0.37 | 0.71 |
| ENSG00000261064 | 0.97 | 0.76 | 1.24 | -0.23 | 0.82 |
| ENSG00000232671 | 0.85 | 0.63 | 1.14 | -1.11 | 0.27 |
| ENSG00000174680 | 0.75 | 0.54 | 1.05 | -1.69 | 0.092 |
| ENSG00000253853 | 0.89 | 0.67 | 1.2 | -0.76 | 0.45 |
| ENSG00000269427 | 1 | 0.77 | 1.31 | 0.01 | 0.99 |
| ENSG00000249378 | 1.14 | 0.87 | 1.48 | 0.95 | 0.34 |
| ENSG00000250331 | 0.89 | 0.65 | 1.2 | -0.77 | 0.44 |
| ENSG00000244968 | 0.75 | 0.55 | 1.02 | -1.84 | 0.065 |
| ENSG00000257354 | 0.79 | 0.6 | 1.04 | -1.65 | 0.098 |
| ENSG00000268087 | 0.94 | 0.71 | 1.24 | -0.44 | 0.66 |
| ENSG00000244513 | 0.8 | 0.62 | 1.05 | -1.61 | 0.11 |
| ENSG00000249906 | 0.67 | 0.43 | 1.03 | -1.81 | 0.071 |
| ENSG00000258789 | 0.86 | 0.65 | 1.15 | -1.01 | 0.31 |
| ENSG00000273784 | 0.72 | 0.54 | 0.96 | -2.26 | 0.024 |
| ENSG00000267454 | 0.85 | 0.65 | 1.13 | -1.12 | 0.26 |
| ENSG00000272273 | 0.83 | 0.65 | 1.07 | -1.4 | 0.16 |
| ENSG00000258824 | 1.03 | 0.79 | 1.34 | 0.23 | 0.82 |
| ENSG00000246465 | 0.97 | 0.77 | 1.24 | -0.21 | 0.84 |
| ENSG00000238117 | 0.94 | 0.74 | 1.19 | -0.51 | 0.61 |
| ENSG00000262995 | 0.84 | 0.42 | 1.66 | -0.51 | 0.61 |
| ENSG00000246523 | 1.02 | 0.79 | 1.31 | 0.15 | 0.88 |
| ENSG00000263684 | 0.67 | 0.37 | 1.21 | -1.33 | 0.18 |
| ENSG00000257259 | 0.99 | 0.8 | 1.23 | -0.07 | 0.94 |
| ENSG00000225376 | 0.97 | 0.74 | 1.28 | -0.21 | 0.83 |
| ENSG00000260693 | 0.86 | 0.65 | 1.13 | -1.09 | 0.28 |
| ENSG00000267683 | 0.89 | 0.62 | 1.27 | -0.66 | 0.51 |
| ENSG00000253105 | 1.2 | 1.01 | 1.43 | 2.09 | 0.037 |
| ENSG00000243220 | 0.8 | 0.47 | 1.38 | -0.8 | 0.42 |
| ENSG00000257327 | 0.83 | 0.63 | 1.1 | -1.29 | 0.2 |
| ENSG00000213373 | 0.53 | 0.24 | 1.19 | -1.54 | 0.12 |
| ENSG00000267097 | 0.64 | 0.39 | 1.05 | -1.77 | 0.076 |
| ENSG00000261480 | 0.7 | 0.47 | 1.03 | -1.83 | 0.067 |
| ENSG00000261218 | 0.88 | 0.64 | 1.2 | -0.81 | 0.42 |
| ENSG00000234184 | 0.92 | 0.68 | 1.25 | -0.55 | 0.58 |
| ENSG00000249096 | 0.96 | 0.72 | 1.28 | -0.26 | 0.79 |
| ENSG00000234883 | 0.83 | 0.62 | 1.11 | -1.23 | 0.22 |
| ENSG00000236935 | 0.69 | 0.49 | 0.99 | -1.99 | 0.046 |
| ENSG00000229151 | 0.89 | 0.66 | 1.19 | -0.81 | 0.42 |
| ENSG00000268804 | 0.79 | 0.54 | 1.15 | -1.23 | 0.22 |
| ENSG00000226777 | 0.93 | 0.69 | 1.27 | -0.44 | 0.66 |
| ENSG00000229228 | 0.83 | 0.59 | 1.15 | -1.12 | 0.26 |
| ENSG00000260719 | 1 | 0.78 | 1.29 | 0 | 1 |
| ENSG00000258987 | 1.12 | 0.91 | 1.37 | 1.04 | 0.3 |
| ENSG00000230943 | 0.92 | 0.71 | 1.2 | -0.6 | 0.55 |
| ENSG00000261211 | 0.89 | 0.67 | 1.17 | -0.83 | 0.4 |
| ENSG00000254864 | 0.79 | 0.6 | 1.05 | -1.62 | 0.1 |
| ENSG00000253361 | 0.94 | 0.72 | 1.22 | -0.45 | 0.65 |
| ENSG00000275484 | 0.86 | 0.65 | 1.14 | -1.05 | 0.29 |
| ENSG00000266947 | 0.93 | 0.71 | 1.2 | -0.56 | 0.58 |
| ENSG00000228624 | 0.98 | 0.72 | 1.34 | -0.1 | 0.92 |
| ENSG00000267666 | 0.92 | 0.7 | 1.22 | -0.57 | 0.57 |
| ENSG00000261742 | 1.07 | 0.83 | 1.37 | 0.49 | 0.62 |
| ENSG00000261039 | 1.07 | 0.83 | 1.39 | 0.53 | 0.6 |
| ENSG00000224854 | 0.98 | 0.73 | 1.31 | -0.14 | 0.89 |
| ENSG00000269353 | 0.91 | 0.67 | 1.26 | -0.55 | 0.58 |
| ENSG00000229109 | 0.89 | 0.69 | 1.16 | -0.84 | 0.4 |
| ENSG00000249203 | 0.85 | 0.64 | 1.12 | -1.18 | 0.24 |
| ENSG00000257337 | 0.74 | 0.56 | 0.99 | -2.02 | 0.043 |
| ENSG00000223784 | 1.17 | 0.98 | 1.39 | 1.78 | 0.075 |
| ENSG00000259495 | 0.94 | 0.72 | 1.22 | -0.49 | 0.62 |
| ENSG00000280119 | 0.85 | 0.65 | 1.1 | -1.27 | 0.2 |
| ENSG00000272933 | 0.82 | 0.64 | 1.05 | -1.56 | 0.12 |
| ENSG00000237685 | 0.97 | 0.71 | 1.32 | -0.21 | 0.84 |
| ENSG00000225208 | 0.78 | 0.58 | 1.06 | -1.58 | 0.11 |
| ENSG00000235978 | 0.96 | 0.74 | 1.24 | -0.34 | 0.74 |
| ENSG00000232194 | 1.18 | 0.88 | 1.59 | 1.12 | 0.26 |
| ENSG00000245213 | 1.03 | 0.79 | 1.35 | 0.25 | 0.81 |
| ENSG00000232415 | 0.74 | 0.55 | 1 | -1.94 | 0.053 |
| ENSG00000280332 | 0.69 | 0.52 | 0.92 | -2.54 | 0.011 |
| ENSG00000276116 | 0.74 | 0.5 | 1.09 | -1.54 | 0.12 |
| ENSG00000225243 | 1.18 | 0.99 | 1.41 | 1.89 | 0.059 |
| ENSG00000272021 | 1.22 | 1.04 | 1.44 | 2.43 | 0.015 |
| ENSG00000276107 | 1.01 | 0.8 | 1.28 | 0.11 | 0.91 |
| ENSG00000233081 | 1.03 | 0.8 | 1.32 | 0.2 | 0.84 |
| ENSG00000260793 | 0.75 | 0.56 | 0.99 | -2.04 | 0.041 |
| ENSG00000237940 | 0.92 | 0.69 | 1.24 | -0.52 | 0.6 |
| ENSG00000213057 | 0.87 | 0.66 | 1.15 | -0.96 | 0.34 |
| ENSG00000260912 | 0.85 | 0.68 | 1.08 | -1.32 | 0.19 |
| ENSG00000277010 | 1.03 | 0.81 | 1.31 | 0.24 | 0.81 |
| ENSG00000266256 | 0.84 | 0.62 | 1.12 | -1.19 | 0.24 |
| ENSG00000280734 | 1.04 | 0.82 | 1.31 | 0.28 | 0.78 |
| ENSG00000279970 | 0.77 | 0.58 | 1.02 | -1.84 | 0.066 |
| ENSG00000248779 | 0.84 | 0.63 | 1.12 | -1.19 | 0.23 |
| ENSG00000241054 | 0.94 | 0.7 | 1.26 | -0.42 | 0.67 |
| ENSG00000251161 | 0.91 | 0.69 | 1.22 | -0.62 | 0.54 |
| ENSG00000272476 | 1.06 | 0.85 | 1.33 | 0.54 | 0.59 |
| ENSG00000237576 | 0.82 | 0.57 | 1.2 | -1.02 | 0.31 |
| ENSG00000271390 | 0.85 | 0.62 | 1.18 | -0.96 | 0.34 |
| ENSG00000253686 | 0.9 | 0.66 | 1.25 | -0.62 | 0.54 |
| ENSG00000264589 | 0.65 | 0.48 | 0.88 | -2.78 | 0.0055 |
| ENSG00000273341 | 0.89 | 0.67 | 1.19 | -0.8 | 0.43 |
| ENSG00000245648 | 0.94 | 0.67 | 1.32 | -0.37 | 0.71 |
| ENSG00000235529 | 1.18 | 0.95 | 1.48 | 1.47 | 0.14 |
| ENSG00000266573 | 0.97 | 0.77 | 1.23 | -0.23 | 0.82 |
| ENSG00000253641 | 1.08 | 0.82 | 1.42 | 0.56 | 0.57 |
| ENSG00000267080 | 0.72 | 0.57 | 0.92 | -2.63 | 0.0087 |
| ENSG00000228290 | 1.12 | 0.86 | 1.48 | 0.83 | 0.4 |
| ENSG00000273055 | 0.77 | 0.55 | 1.07 | -1.55 | 0.12 |
| ENSG00000258100 | 0.84 | 0.63 | 1.14 | -1.1 | 0.27 |
| ENSG00000236711 | 0.74 | 0.54 | 1.02 | -1.85 | 0.064 |
| ENSG00000259124 | 0.9 | 0.67 | 1.19 | -0.75 | 0.45 |
| ENSG00000233461 | 0.8 | 0.61 | 1.06 | -1.55 | 0.12 |
| ENSG00000267082 | 0.79 | 0.59 | 1.05 | -1.65 | 0.1 |
| ENSG00000249923 | 0.93 | 0.7 | 1.23 | -0.5 | 0.62 |
| ENSG00000259887 | 1.11 | 0.88 | 1.41 | 0.91 | 0.36 |
| ENSG00000242908 | 0.96 | 0.74 | 1.25 | -0.27 | 0.78 |
| ENSG00000237380 | 0.75 | 0.54 | 1.05 | -1.67 | 0.095 |
| ENSG00000253702 | 0.95 | 0.71 | 1.27 | -0.36 | 0.72 |
| ENSG00000267280 | 1.09 | 0.83 | 1.42 | 0.63 | 0.53 |
| ENSG00000274719 | 1.05 | 0.81 | 1.36 | 0.39 | 0.7 |
| ENSG00000278383 | 1.07 | 0.83 | 1.38 | 0.52 | 0.6 |
| ENSG00000261399 | 0.89 | 0.67 | 1.2 | -0.76 | 0.45 |
| ENSG00000267714 | 0.68 | 0.41 | 1.11 | -1.56 | 0.12 |
| ENSG00000255644 | 0.92 | 0.67 | 1.27 | -0.48 | 0.63 |
| ENSG00000224417 | 0.61 | 0.28 | 1.36 | -1.2 | 0.23 |
| ENSG00000236708 | 1.09 | 0.83 | 1.44 | 0.61 | 0.54 |
| ENSG00000237234 | 1.02 | 0.78 | 1.35 | 0.18 | 0.86 |
| ENSG00000177822 | 0.98 | 0.77 | 1.24 | -0.19 | 0.85 |
| ENSG00000231024 | 1.3 | 1.03 | 1.65 | 2.19 | 0.028 |
| ENSG00000267650 | 1.29 | 0.98 | 1.7 | 1.8 | 0.073 |
| ENSG00000205231 | 0.87 | 0.67 | 1.13 | -1.05 | 0.29 |
| ENSG00000234678 | 1.01 | 0.78 | 1.3 | 0.08 | 0.94 |
| ENSG00000259153 | 0.87 | 0.66 | 1.13 | -1.05 | 0.29 |
| ENSG00000272425 | 1.1 | 0.88 | 1.39 | 0.86 | 0.39 |
| ENSG00000183674 | 1.15 | 0.91 | 1.46 | 1.17 | 0.24 |
| ENSG00000237751 | 0.87 | 0.63 | 1.21 | -0.83 | 0.41 |
| ENSG00000233725 | 1.12 | 0.96 | 1.3 | 1.45 | 0.15 |
| ENSG00000263588 | 0.96 | 0.75 | 1.23 | -0.3 | 0.76 |
| ENSG00000224717 | 1.04 | 0.81 | 1.34 | 0.32 | 0.75 |
| ENSG00000232598 | 0.75 | 0.48 | 1.15 | -1.33 | 0.18 |
| ENSG00000224666 | 0.87 | 0.65 | 1.16 | -0.95 | 0.34 |
| ENSG00000238266 | 1.05 | 0.87 | 1.26 | 0.5 | 0.62 |
| ENSG00000253196 | 1.16 | 0.88 | 1.53 | 1.02 | 0.31 |
| ENSG00000253217 | 1.05 | 0.86 | 1.29 | 0.49 | 0.62 |
| ENSG00000234753 | 1.03 | 0.82 | 1.3 | 0.28 | 0.78 |
| ENSG00000234506 | 1.05 | 0.82 | 1.34 | 0.39 | 0.7 |
| ENSG00000254288 | 0.9 | 0.69 | 1.19 | -0.74 | 0.46 |
| ENSG00000225518 | 0.87 | 0.67 | 1.12 | -1.09 | 0.28 |
| ENSG00000261105 | 0.92 | 0.7 | 1.21 | -0.6 | 0.55 |
| ENSG00000237923 | 0.85 | 0.63 | 1.14 | -1.11 | 0.27 |
| ENSG00000265487 | 0.79 | 0.58 | 1.09 | -1.44 | 0.15 |
| ENSG00000280423 | 1.02 | 0.81 | 1.3 | 0.19 | 0.85 |
| ENSG00000267466 | 0.92 | 0.7 | 1.2 | -0.64 | 0.52 |
| ENSG00000273261 | 0.96 | 0.75 | 1.24 | -0.28 | 0.78 |
| ENSG00000259070 | 0.95 | 0.75 | 1.2 | -0.46 | 0.65 |
| ENSG00000206567 | 0.9 | 0.68 | 1.2 | -0.71 | 0.48 |
| ENSG00000259985 | 0.98 | 0.77 | 1.24 | -0.18 | 0.86 |
| ENSG00000260552 | 0.72 | 0.53 | 0.98 | -2.08 | 0.038 |
| ENSG00000256234 | 0.67 | 0.47 | 0.94 | -2.3 | 0.021 |
| ENSG00000276529 | 1.05 | 0.82 | 1.36 | 0.41 | 0.68 |
| ENSG00000198221 | 0.96 | 0.74 | 1.23 | -0.35 | 0.73 |
| ENSG00000236856 | 0.56 | 0.25 | 1.24 | -1.43 | 0.15 |
| ENSG00000246334 | 1.02 | 0.8 | 1.31 | 0.19 | 0.85 |
| ENSG00000260015 | 1.11 | 0.94 | 1.31 | 1.25 | 0.21 |
| ENSG00000236145 | 0.87 | 0.58 | 1.3 | -0.68 | 0.5 |
| ENSG00000267506 | 0.86 | 0.62 | 1.2 | -0.89 | 0.38 |
| ENSG00000224509 | 1.32 | 1.08 | 1.61 | 2.74 | 0.0062 |
| ENSG00000234477 | 1.27 | 1.03 | 1.57 | 2.21 | 0.027 |
| ENSG00000260420 | 1.24 | 1.03 | 1.5 | 2.29 | 0.022 |
| ENSG00000267432 | 0.95 | 0.74 | 1.22 | -0.4 | 0.69 |
| ENSG00000205334 | 0.9 | 0.63 | 1.28 | -0.59 | 0.55 |
| ENSG00000273214 | 0.39 | 0.13 | 1.16 | -1.69 | 0.092 |
| ENSG00000258077 | 1.32 | 1.07 | 1.63 | 2.62 | 0.0089 |
| ENSG00000241155 | 0.91 | 0.7 | 1.19 | -0.71 | 0.48 |
| ENSG00000273321 | 0.93 | 0.71 | 1.21 | -0.56 | 0.57 |
| ENSG00000277463 | 1.16 | 0.96 | 1.41 | 1.53 | 0.13 |
| ENSG00000234476 | 0.77 | 0.46 | 1.26 | -1.05 | 0.3 |
| ENSG00000254626 | 1.07 | 0.82 | 1.39 | 0.51 | 0.61 |
| ENSG00000231764 | 0.97 | 0.73 | 1.3 | -0.2 | 0.84 |
| ENSG00000278200 | 1.16 | 0.95 | 1.41 | 1.49 | 0.14 |
| ENSG00000236508 | 1.06 | 0.82 | 1.38 | 0.43 | 0.67 |
| ENSG00000263893 | 1.01 | 0.79 | 1.28 | 0.05 | 0.96 |
| ENSG00000232656 | 1.08 | 0.92 | 1.26 | 0.95 | 0.34 |
| ENSG00000206249 | 0.85 | 0.64 | 1.13 | -1.11 | 0.27 |
| ENSG00000266936 | 1.05 | 0.84 | 1.31 | 0.45 | 0.65 |
| ENSG00000259230 | 0.96 | 0.78 | 1.18 | -0.4 | 0.69 |
| ENSG00000223695 | 1.12 | 0.89 | 1.42 | 0.96 | 0.34 |
| ENSG00000271659 | 0.99 | 0.78 | 1.26 | -0.07 | 0.94 |
| ENSG00000230090 | 0.86 | 0.63 | 1.18 | -0.93 | 0.35 |
| ENSG00000269495 | 1.14 | 0.98 | 1.32 | 1.64 | 0.1 |
| ENSG00000261786 | 0.79 | 0.55 | 1.13 | -1.31 | 0.19 |
| ENSG00000279949 | 0.99 | 0.75 | 1.3 | -0.08 | 0.94 |
| ENSG00000268601 | 1.17 | 0.96 | 1.44 | 1.54 | 0.12 |
| ENSG00000233682 | 1.04 | 0.81 | 1.34 | 0.32 | 0.75 |
| ENSG00000245248 | 1.09 | 0.89 | 1.33 | 0.8 | 0.43 |
| ENSG00000272431 | 0.99 | 0.78 | 1.26 | -0.08 | 0.94 |
| ENSG00000205325 | 0.9 | 0.66 | 1.22 | -0.68 | 0.49 |
| ENSG00000266278 | 0.97 | 0.72 | 1.32 | -0.17 | 0.87 |
| ENSG00000260400 | 0.99 | 0.76 | 1.29 | -0.05 | 0.96 |
| ENSG00000245571 | 0.9 | 0.71 | 1.15 | -0.83 | 0.41 |
| ENSG00000276223 | 0.95 | 0.73 | 1.22 | -0.42 | 0.68 |
| ENSG00000266913 | 0.99 | 0.74 | 1.32 | -0.07 | 0.94 |
| ENSG00000255727 | 0.71 | 0.53 | 0.95 | -2.27 | 0.023 |
| ENSG00000259408 | 0.96 | 0.74 | 1.25 | -0.29 | 0.77 |
| ENSG00000232973 | 0.93 | 0.7 | 1.23 | -0.53 | 0.6 |
| ENSG00000228288 | 1.04 | 0.82 | 1.33 | 0.33 | 0.74 |
| ENSG00000250073 | 1.06 | 0.85 | 1.33 | 0.51 | 0.61 |
| ENSG00000231651 | 0.93 | 0.68 | 1.27 | -0.47 | 0.64 |
| ENSG00000167920 | 0.76 | 0.59 | 0.98 | -2.12 | 0.034 |
| ENSG00000260876 | 0.95 | 0.72 | 1.24 | -0.4 | 0.69 |
| ENSG00000240040 | 0.95 | 0.75 | 1.2 | -0.46 | 0.64 |
| ENSG00000276980 | 0.88 | 0.68 | 1.12 | -1.04 | 0.3 |
| ENSG00000263325 | 0.99 | 0.76 | 1.29 | -0.08 | 0.94 |
| ENSG00000267304 | 0.7 | 0.42 | 1.15 | -1.43 | 0.15 |
| ENSG00000224204 | 1.1 | 0.95 | 1.26 | 1.28 | 0.2 |
| ENSG00000230461 | 1.05 | 0.69 | 1.61 | 0.24 | 0.81 |
| ENSG00000228222 | 0.95 | 0.64 | 1.41 | -0.26 | 0.8 |
| ENSG00000258851 | 0.94 | 0.68 | 1.29 | -0.4 | 0.69 |
| ENSG00000262061 | 1.09 | 0.91 | 1.3 | 0.97 | 0.33 |
| ENSG00000267440 | 1 | 0.81 | 1.24 | -0.01 | 0.99 |
| ENSG00000230730 | 0.99 | 0.76 | 1.3 | -0.06 | 0.95 |
| ENSG00000254863 | 0.91 | 0.64 | 1.29 | -0.53 | 0.6 |
| ENSG00000272784 | 0.82 | 0.61 | 1.09 | -1.39 | 0.16 |
| ENSG00000274220 | 1.16 | 0.91 | 1.49 | 1.21 | 0.23 |
| ENSG00000233754 | 1.08 | 0.9 | 1.3 | 0.85 | 0.4 |
| ENSG00000272950 | 0.96 | 0.76 | 1.2 | -0.39 | 0.7 |
| ENSG00000229019 | 0.88 | 0.67 | 1.16 | -0.89 | 0.37 |
| ENSG00000234902 | 0.97 | 0.75 | 1.27 | -0.19 | 0.85 |
| ENSG00000248339 | 1.03 | 0.74 | 1.44 | 0.18 | 0.86 |
| ENSG00000253389 | 0.84 | 0.45 | 1.55 | -0.57 | 0.57 |
| ENSG00000254254 | 1.23 | 0.71 | 2.13 | 0.75 | 0.45 |
| ENSG00000258231 | 1.08 | 0.95 | 1.24 | 1.17 | 0.24 |
| ENSG00000249742 | 0.9 | 0.6 | 1.34 | -0.54 | 0.59 |
| ENSG00000229491 | 1.09 | 0.85 | 1.4 | 0.7 | 0.48 |
| ENSG00000271820 | 0.87 | 0.63 | 1.21 | -0.82 | 0.41 |
| ENSG00000234076 | 0.85 | 0.64 | 1.12 | -1.13 | 0.26 |
| ENSG00000254302 | 0.93 | 0.69 | 1.25 | -0.49 | 0.62 |
| ENSG00000225205 | 0.91 | 0.68 | 1.21 | -0.65 | 0.52 |
| ENSG00000275719 | 0.99 | 0.75 | 1.32 | -0.04 | 0.97 |
| ENSG00000225439 | 0.91 | 0.7 | 1.18 | -0.74 | 0.46 |
| ENSG00000230314 | 0.76 | 0.57 | 1.01 | -1.87 | 0.062 |
| ENSG00000259977 | 0.92 | 0.73 | 1.16 | -0.7 | 0.49 |
| ENSG00000235688 | 1.09 | 0.82 | 1.45 | 0.6 | 0.55 |
| ENSG00000257869 | 1.3 | 1.01 | 1.66 | 2.06 | 0.039 |
| ENSG00000273153 | 0.84 | 0.62 | 1.13 | -1.14 | 0.25 |
| ENSG00000196366 | 1.14 | 0.88 | 1.5 | 0.99 | 0.32 |
| ENSG00000248690 | 1.04 | 0.82 | 1.31 | 0.31 | 0.76 |
| ENSG00000280068 | 1.22 | 1 | 1.49 | 1.96 | 0.05 |
| ENSG00000235314 | 0.99 | 0.76 | 1.29 | -0.09 | 0.93 |
| ENSG00000237594 | 1.06 | 0.83 | 1.35 | 0.45 | 0.65 |
| ENSG00000176912 | 0.97 | 0.73 | 1.3 | -0.18 | 0.86 |
| ENSG00000232445 | 1.12 | 0.86 | 1.46 | 0.83 | 0.41 |
| ENSG00000274340 | 0.79 | 0.6 | 1.04 | -1.7 | 0.089 |
| ENSG00000203362 | 1.01 | 0.8 | 1.27 | 0.05 | 0.96 |
| ENSG00000251450 | 0.89 | 0.64 | 1.24 | -0.69 | 0.49 |
| ENSG00000230148 | 0.71 | 0.53 | 0.95 | -2.35 | 0.019 |
| ENSG00000272908 | 1.09 | 0.87 | 1.36 | 0.75 | 0.45 |
| ENSG00000237928 | 0.78 | 0.55 | 1.1 | -1.42 | 0.16 |
| ENSG00000265485 | 1.1 | 0.96 | 1.26 | 1.34 | 0.18 |
| ENSG00000232679 | 1.2 | 0.94 | 1.52 | 1.49 | 0.14 |
| ENSG00000249001 | 1.13 | 0.87 | 1.46 | 0.89 | 0.37 |
| ENSG00000275418 | 0.94 | 0.73 | 1.22 | -0.44 | 0.66 |
| ENSG00000235532 | 0.92 | 0.68 | 1.23 | -0.56 | 0.57 |
| ENSG00000229953 | 1.02 | 0.8 | 1.31 | 0.16 | 0.87 |
| ENSG00000273837 | 0.94 | 0.72 | 1.22 | -0.48 | 0.63 |
| ENSG00000224965 | 1.02 | 0.81 | 1.29 | 0.17 | 0.86 |
| ENSG00000243795 | 0.93 | 0.69 | 1.25 | -0.48 | 0.63 |
| ENSG00000224950 | 0.86 | 0.64 | 1.14 | -1.04 | 0.3 |
| ENSG00000234199 | 1.12 | 0.94 | 1.34 | 1.29 | 0.2 |
| ENSG00000279965 | 0.69 | 0.4 | 1.18 | -1.35 | 0.18 |
| ENSG00000244676 | 0.98 | 0.77 | 1.24 | -0.2 | 0.84 |
| ENSG00000258512 | 0.85 | 0.65 | 1.13 | -1.1 | 0.27 |
| ENSG00000250509 | 0.76 | 0.49 | 1.2 | -1.18 | 0.24 |
| ENSG00000272155 | 0.86 | 0.65 | 1.13 | -1.09 | 0.28 |
| ENSG00000275465 | 0.79 | 0.47 | 1.34 | -0.87 | 0.38 |
| ENSG00000224251 | 1 | 0.8 | 1.26 | 0.01 | 0.99 |
| ENSG00000277301 | 0.95 | 0.73 | 1.23 | -0.39 | 0.69 |
| ENSG00000227617 | 0.84 | 0.61 | 1.15 | -1.1 | 0.27 |
| ENSG00000228630 | 1.27 | 1 | 1.61 | 1.94 | 0.052 |
| ENSG00000254826 | 1.17 | 0.9 | 1.51 | 1.19 | 0.23 |
| ENSG00000254528 | 1.04 | 0.83 | 1.31 | 0.34 | 0.73 |
| ENSG00000249087 | 0.89 | 0.67 | 1.2 | -0.75 | 0.45 |
| ENSG00000277863 | 0.82 | 0.61 | 1.11 | -1.26 | 0.21 |
| ENSG00000093100 | 0.95 | 0.71 | 1.26 | -0.38 | 0.7 |
| ENSG00000271976 | 0.9 | 0.69 | 1.18 | -0.78 | 0.44 |
| ENSG00000226581 | 1.02 | 0.8 | 1.31 | 0.2 | 0.84 |
| ENSG00000230074 | 0.87 | 0.67 | 1.13 | -1.04 | 0.3 |
| ENSG00000186235 | 1.08 | 0.86 | 1.34 | 0.64 | 0.53 |
| ENSG00000248925 | 1.06 | 0.85 | 1.32 | 0.5 | 0.62 |
| ENSG00000258943 | 1.06 | 0.84 | 1.32 | 0.47 | 0.64 |
| ENSG00000258940 | 0.93 | 0.71 | 1.21 | -0.56 | 0.58 |
| ENSG00000270457 | 0.87 | 0.69 | 1.1 | -1.17 | 0.24 |
| ENSG00000274624 | 0.83 | 0.62 | 1.11 | -1.25 | 0.21 |
| ENSG00000255176 | 0.92 | 0.7 | 1.22 | -0.56 | 0.57 |
| ENSG00000226440 | 0.95 | 0.7 | 1.28 | -0.34 | 0.73 |
| ENSG00000232527 | 0.85 | 0.61 | 1.18 | -0.99 | 0.32 |
| ENSG00000188242 | 1.07 | 0.83 | 1.37 | 0.49 | 0.63 |
| ENSG00000235782 | 0.96 | 0.74 | 1.24 | -0.33 | 0.74 |
| ENSG00000257660 | 0.9 | 0.68 | 1.18 | -0.79 | 0.43 |
| ENSG00000280417 | 0.92 | 0.72 | 1.18 | -0.64 | 0.52 |
| ENSG00000261416 | 0.92 | 0.7 | 1.22 | -0.55 | 0.58 |
| ENSG00000272463 | 0.61 | 0.43 | 0.86 | -2.8 | 0.0051 |
| ENSG00000238164 | 0.67 | 0.49 | 0.9 | -2.65 | 0.008 |
| ENSG00000177640 | 0.83 | 0.64 | 1.08 | -1.41 | 0.16 |
| ENSG00000234362 | 0.87 | 0.62 | 1.24 | -0.76 | 0.45 |
| ENSG00000240086 | 0.89 | 0.67 | 1.18 | -0.82 | 0.41 |
| ENSG00000274922 | 0.75 | 0.55 | 1.04 | -1.73 | 0.084 |
| ENSG00000272279 | 0.83 | 0.62 | 1.11 | -1.27 | 0.2 |
| ENSG00000232721 | 1.12 | 0.95 | 1.32 | 1.31 | 0.19 |
| ENSG00000267745 | 0.81 | 0.62 | 1.04 | -1.64 | 0.1 |
| ENSG00000261338 | 0.99 | 0.78 | 1.25 | -0.1 | 0.92 |
| ENSG00000272666 | 0.78 | 0.59 | 1.03 | -1.74 | 0.082 |
| ENSG00000270021 | 0.93 | 0.72 | 1.2 | -0.57 | 0.57 |
| ENSG00000213904 | 0.74 | 0.54 | 1.03 | -1.77 | 0.077 |
| ENSG00000174365 | 0.87 | 0.69 | 1.11 | -1.11 | 0.27 |
| ENSG00000250091 | 1.06 | 0.84 | 1.34 | 0.48 | 0.63 |
| ENSG00000272975 | 1.12 | 0.89 | 1.4 | 0.94 | 0.35 |
| ENSG00000230266 | 0.9 | 0.66 | 1.22 | -0.68 | 0.49 |
| ENSG00000234663 | 0.83 | 0.6 | 1.14 | -1.16 | 0.25 |
| ENSG00000233355 | 0.96 | 0.7 | 1.3 | -0.29 | 0.77 |
| ENSG00000233387 | 0.91 | 0.69 | 1.21 | -0.63 | 0.53 |
| ENSG00000228037 | 0.79 | 0.59 | 1.07 | -1.52 | 0.13 |
| ENSG00000244620 | 1.01 | 0.75 | 1.37 | 0.09 | 0.93 |
| ENSG00000224220 | 0.85 | 0.63 | 1.17 | -0.99 | 0.32 |
| ENSG00000233308 | 0.77 | 0.53 | 1.13 | -1.32 | 0.19 |
| ENSG00000230630 | 1.17 | 0.89 | 1.53 | 1.15 | 0.25 |
| ENSG00000234779 | 0.87 | 0.63 | 1.22 | -0.81 | 0.42 |
| ENSG00000261076 | 0.95 | 0.72 | 1.25 | -0.38 | 0.7 |
| ENSG00000177757 | 0.79 | 0.58 | 1.07 | -1.55 | 0.12 |
| ENSG00000258548 | 0.91 | 0.51 | 1.64 | -0.3 | 0.76 |
| ENSG00000227352 | 1 | 0.8 | 1.25 | -0.01 | 0.99 |
| ENSG00000247809 | 0.85 | 0.64 | 1.13 | -1.1 | 0.27 |
| ENSG00000231613 | 0.88 | 0.67 | 1.17 | -0.87 | 0.39 |
| ENSG00000251602 | 0.86 | 0.67 | 1.11 | -1.15 | 0.25 |
| ENSG00000197813 | 1.17 | 0.94 | 1.45 | 1.38 | 0.17 |
| ENSG00000261469 | 0.93 | 0.7 | 1.23 | -0.52 | 0.6 |
| ENSG00000249116 | 1.3 | 0.98 | 1.71 | 1.85 | 0.064 |
| ENSG00000238755 | 0.73 | 0.46 | 1.14 | -1.38 | 0.17 |
| ENSG00000267787 | 0.95 | 0.73 | 1.23 | -0.39 | 0.7 |
| ENSG00000177738 | 1.15 | 0.9 | 1.46 | 1.13 | 0.26 |
| ENSG00000253768 | 0.79 | 0.61 | 1.03 | -1.72 | 0.086 |
| ENSG00000279685 | 0.71 | 0.5 | 1 | -1.95 | 0.051 |
| ENSG00000254164 | 0.86 | 0.67 | 1.11 | -1.14 | 0.26 |
| ENSG00000249574 | 1.17 | 0.95 | 1.44 | 1.47 | 0.14 |
| ENSG00000272491 | 0.75 | 0.54 | 1.04 | -1.74 | 0.083 |
| ENSG00000271367 | 0.78 | 0.6 | 1.01 | -1.92 | 0.055 |
| ENSG00000268555 | 0.9 | 0.67 | 1.2 | -0.72 | 0.47 |
| ENSG00000245685 | 1 | 0.71 | 1.4 | -0.02 | 0.99 |
| ENSG00000261659 | 0.87 | 0.65 | 1.16 | -0.95 | 0.34 |
| ENSG00000270607 | 0.74 | 0.52 | 1.06 | -1.64 | 0.1 |
| ENSG00000254744 | 0.86 | 0.64 | 1.16 | -1 | 0.32 |
| ENSG00000229852 | 0.76 | 0.58 | 1 | -1.99 | 0.047 |
| ENSG00000214043 | 0.73 | 0.45 | 1.19 | -1.26 | 0.21 |
| ENSG00000261215 | 0.64 | 0.46 | 0.88 | -2.73 | 0.0064 |
| ENSG00000271758 | 0.56 | 0.27 | 1.16 | -1.55 | 0.12 |
| ENSG00000259170 | 0.71 | 0.46 | 1.12 | -1.48 | 0.14 |
| ENSG00000249125 | 0.96 | 0.71 | 1.3 | -0.24 | 0.81 |
| ENSG00000259928 | 1 | 0.76 | 1.31 | -0.03 | 0.98 |
| ENSG00000224746 | 1.04 | 0.84 | 1.3 | 0.37 | 0.71 |
| ENSG00000253660 | 1.06 | 0.83 | 1.35 | 0.44 | 0.66 |
| ENSG00000235304 | 1.01 | 0.81 | 1.26 | 0.08 | 0.94 |
| ENSG00000266088 | 0.85 | 0.64 | 1.12 | -1.16 | 0.25 |
| ENSG00000257924 | 0.95 | 0.73 | 1.23 | -0.39 | 0.7 |
| ENSG00000229950 | 0.71 | 0.53 | 0.95 | -2.29 | 0.022 |
| ENSG00000259087 | 1.11 | 0.86 | 1.43 | 0.83 | 0.41 |
| ENSG00000267904 | 1.08 | 0.83 | 1.41 | 0.56 | 0.58 |
| ENSG00000265519 | 0.83 | 0.64 | 1.08 | -1.39 | 0.16 |
| ENSG00000260966 | 0.88 | 0.66 | 1.16 | -0.91 | 0.36 |
| ENSG00000269044 | 0.85 | 0.65 | 1.09 | -1.27 | 0.2 |
| ENSG00000268912 | 1 | 0.77 | 1.29 | -0.04 | 0.97 |
| ENSG00000272168 | 1.02 | 0.77 | 1.36 | 0.15 | 0.88 |
| ENSG00000253397 | 0.68 | 0.36 | 1.31 | -1.14 | 0.25 |
| ENSG00000226889 | 0.9 | 0.69 | 1.16 | -0.81 | 0.42 |
| ENSG00000275830 | 0.65 | 0.39 | 1.09 | -1.63 | 0.1 |
| ENSG00000228950 | 0.92 | 0.7 | 1.21 | -0.61 | 0.54 |
| ENSG00000214293 | 0.85 | 0.65 | 1.1 | -1.23 | 0.22 |
| ENSG00000242078 | 0.9 | 0.69 | 1.19 | -0.72 | 0.47 |
| ENSG00000236830 | 1.29 | 0.99 | 1.67 | 1.91 | 0.056 |
| ENSG00000267629 | 0.96 | 0.7 | 1.31 | -0.27 | 0.79 |
| ENSG00000235023 | 1.17 | 0.98 | 1.4 | 1.72 | 0.085 |
| ENSG00000270720 | 0.92 | 0.71 | 1.19 | -0.63 | 0.53 |
| ENSG00000176236 | 0.92 | 0.7 | 1.21 | -0.57 | 0.57 |
| ENSG00000272970 | 0.9 | 0.67 | 1.2 | -0.74 | 0.46 |
| ENSG00000279266 | 1.12 | 0.97 | 1.29 | 1.48 | 0.14 |
| ENSG00000257588 | 1.18 | 0.9 | 1.55 | 1.2 | 0.23 |
| ENSG00000261959 | 0.79 | 0.46 | 1.33 | -0.89 | 0.37 |
| ENSG00000233723 | 0.52 | 0.33 | 0.81 | -2.84 | 0.0045 |
| ENSG00000231527 | 0.99 | 0.77 | 1.28 | -0.06 | 0.95 |
| ENSG00000234928 | 0.96 | 0.74 | 1.25 | -0.29 | 0.77 |
| ENSG00000243230 | 0.79 | 0.58 | 1.07 | -1.52 | 0.13 |
| ENSG00000152931 | 1.22 | 0.98 | 1.53 | 1.77 | 0.077 |
| ENSG00000253414 | 0.95 | 0.72 | 1.25 | -0.38 | 0.7 |
| ENSG00000227531 | 0.85 | 0.6 | 1.2 | -0.93 | 0.35 |
| ENSG00000256039 | 0.64 | 0.45 | 0.93 | -2.37 | 0.018 |
| ENSG00000272625 | 0.87 | 0.65 | 1.18 | -0.88 | 0.38 |
| ENSG00000235029 | 0.93 | 0.69 | 1.24 | -0.52 | 0.6 |
| ENSG00000254319 | 0.99 | 0.73 | 1.35 | -0.04 | 0.97 |
| ENSG00000277351 | 0.97 | 0.72 | 1.3 | -0.23 | 0.82 |
| ENSG00000206187 | 0.83 | 0.49 | 1.39 | -0.71 | 0.48 |
| ENSG00000251061 | 1.08 | 0.88 | 1.33 | 0.77 | 0.44 |
| ENSG00000260166 | 1.08 | 0.86 | 1.35 | 0.65 | 0.51 |
| ENSG00000227082 | 0.92 | 0.67 | 1.26 | -0.54 | 0.59 |
| ENSG00000267405 | 0.76 | 0.51 | 1.12 | -1.39 | 0.16 |
| ENSG00000243818 | 1.07 | 0.82 | 1.4 | 0.51 | 0.61 |
| ENSG00000229740 | 0.89 | 0.61 | 1.28 | -0.65 | 0.52 |
| ENSG00000274080 | 0.84 | 0.61 | 1.16 | -1.04 | 0.3 |
| ENSG00000248428 | 0.94 | 0.76 | 1.15 | -0.63 | 0.53 |
| ENSG00000272564 | 0.92 | 0.69 | 1.21 | -0.61 | 0.54 |
| ENSG00000246263 | 0.98 | 0.76 | 1.27 | -0.13 | 0.9 |
| ENSG00000244558 | 1.08 | 0.86 | 1.36 | 0.65 | 0.52 |
| ENSG00000227857 | 1.13 | 0.88 | 1.45 | 0.94 | 0.34 |
| ENSG00000214106 | 0.94 | 0.73 | 1.22 | -0.44 | 0.66 |
| ENSG00000261189 | 1.08 | 0.85 | 1.37 | 0.63 | 0.53 |
| ENSG00000225383 | 1.15 | 0.92 | 1.44 | 1.27 | 0.2 |
| ENSG00000234261 | 1.3 | 1.03 | 1.63 | 2.22 | 0.026 |
| ENSG00000271778 | 0.73 | 0.54 | 0.98 | -2.13 | 0.034 |
| ENSG00000213062 | 0.89 | 0.69 | 1.15 | -0.9 | 0.37 |
| ENSG00000254670 | 0.99 | 0.77 | 1.29 | -0.05 | 0.96 |
| ENSG00000271040 | 1.02 | 0.78 | 1.32 | 0.12 | 0.9 |
| ENSG00000257989 | 1.19 | 0.97 | 1.46 | 1.63 | 0.1 |
| ENSG00000258871 | 1.03 | 0.81 | 1.33 | 0.27 | 0.79 |
| ENSG00000260439 | 0.88 | 0.67 | 1.15 | -0.96 | 0.34 |
| ENSG00000260686 | 0.92 | 0.7 | 1.19 | -0.65 | 0.51 |
| ENSG00000254731 | 0.88 | 0.65 | 1.21 | -0.78 | 0.43 |
| ENSG00000255250 | 0.62 | 0.41 | 0.94 | -2.23 | 0.025 |
| ENSG00000254854 | 1.1 | 0.86 | 1.42 | 0.78 | 0.43 |
| ENSG00000247287 | 0.91 | 0.69 | 1.19 | -0.7 | 0.48 |
| ENSG00000259345 | 1.12 | 0.91 | 1.38 | 1.08 | 0.28 |
| ENSG00000249641 | 1.08 | 0.85 | 1.38 | 0.66 | 0.51 |
| ENSG00000251151 | 1.02 | 0.79 | 1.32 | 0.17 | 0.86 |
| ENSG00000261644 | 0.92 | 0.71 | 1.2 | -0.63 | 0.53 |
| ENSG00000251632 | 0.77 | 0.35 | 1.68 | -0.66 | 0.51 |
| ENSG00000250126 | 0.92 | 0.66 | 1.3 | -0.46 | 0.65 |
| ENSG00000254404 | 0.78 | 0.56 | 1.1 | -1.4 | 0.16 |
| ENSG00000270179 | 0.97 | 0.76 | 1.23 | -0.27 | 0.79 |
| ENSG00000255224 | 1.04 | 0.8 | 1.34 | 0.26 | 0.79 |
| ENSG00000255733 | 0.92 | 0.69 | 1.23 | -0.56 | 0.57 |
| ENSG00000260997 | 0.72 | 0.52 | 0.99 | -2.01 | 0.045 |
| ENSG00000224137 | 0.94 | 0.72 | 1.22 | -0.47 | 0.64 |
| ENSG00000279588 | 0.9 | 0.69 | 1.16 | -0.84 | 0.4 |
| ENSG00000259577 | 0.87 | 0.65 | 1.17 | -0.92 | 0.36 |
| ENSG00000226026 | 0.8 | 0.59 | 1.09 | -1.42 | 0.16 |
| ENSG00000229589 | 0.74 | 0.56 | 0.98 | -2.09 | 0.037 |
| ENSG00000253988 | 1.08 | 0.82 | 1.42 | 0.54 | 0.59 |
| ENSG00000259353 | 1.25 | 0.98 | 1.58 | 1.83 | 0.067 |
| ENSG00000241220 | 0.85 | 0.62 | 1.18 | -0.97 | 0.33 |
| ENSG00000271964 | 0.77 | 0.58 | 1.02 | -1.83 | 0.067 |
| ENSG00000260941 | 1.01 | 0.86 | 1.18 | 0.11 | 0.91 |
| ENSG00000260954 | 0.76 | 0.56 | 1.04 | -1.74 | 0.083 |
| ENSG00000260989 | 0.87 | 0.65 | 1.16 | -0.96 | 0.34 |
| ENSG00000229692 | 1.02 | 0.8 | 1.3 | 0.15 | 0.88 |
| ENSG00000261889 | 0.75 | 0.56 | 0.99 | -2 | 0.046 |
| ENSG00000261465 | 1.16 | 0.84 | 1.59 | 0.88 | 0.38 |
| ENSG00000263072 | 0.87 | 0.68 | 1.1 | -1.17 | 0.24 |
| ENSG00000260852 | 0.96 | 0.75 | 1.24 | -0.3 | 0.76 |
| ENSG00000280132 | 0.86 | 0.64 | 1.16 | -0.98 | 0.32 |
| ENSG00000275155 | 0.98 | 0.73 | 1.31 | -0.17 | 0.87 |
| ENSG00000246526 | 0.94 | 0.72 | 1.24 | -0.41 | 0.68 |
| ENSG00000259802 | 1.04 | 0.8 | 1.36 | 0.32 | 0.75 |
| ENSG00000234636 | 0.82 | 0.61 | 1.1 | -1.35 | 0.18 |
| ENSG00000243018 | 0.74 | 0.54 | 1.02 | -1.84 | 0.065 |
| ENSG00000241593 | 0.77 | 0.48 | 1.23 | -1.1 | 0.27 |
| ENSG00000251381 | 0.86 | 0.64 | 1.17 | -0.96 | 0.34 |
| ENSG00000262884 | 1.19 | 0.92 | 1.55 | 1.32 | 0.19 |
| ENSG00000255507 | 1.11 | 0.87 | 1.43 | 0.85 | 0.4 |
| ENSG00000248464 | 1.09 | 0.92 | 1.29 | 0.98 | 0.33 |
| ENSG00000267299 | 0.84 | 0.63 | 1.12 | -1.16 | 0.25 |
| ENSG00000247970 | 0.93 | 0.7 | 1.23 | -0.53 | 0.6 |
| ENSG00000166917 | 0.77 | 0.55 | 1.08 | -1.52 | 0.13 |
| ENSG00000231324 | 1.21 | 1 | 1.47 | 1.99 | 0.047 |
| ENSG00000232316 | 0.95 | 0.73 | 1.23 | -0.41 | 0.68 |
| ENSG00000224271 | 1.35 | 1.1 | 1.66 | 2.82 | 0.0047 |
