# Supplementary material for: Identification and validation of an epigenetically regulated long noncoding RNA model for breast cancer metabolism and prognosis
Source: BMC Med Genomics. 2022 May 7;15:105. doi: 10.1186/s12920-022-01256-2 (PMC9077958; doi:10.1186/s12920-022-01256-2)
Supplement: Supplementary file 5 — Additional file 5: Table S5. The 86 KEGG pathways correlated with tumor metabolism. [file 12920_2022_1256_MOESM5_ESM.zip › Supplementary_table_S5.docx]

**Table S5. The 86 KEGG pathways correlated with tumor metabolism.**

| KEGG pathway | pathway class | regulation | logFC | P.Value | adj.P.Val |
| --- | --- | --- | --- | --- | --- |
| Glycolysis / Gluconeogenesis | Carbohydrate metabolism | Up | 0.107596 | 4.12E-19 | 7.09E-18 |
| Citrate cycle (TCA cycle) | Carbohydrate metabolism | Up | 0.172408 | 1.70E-20 | 3.66E-19 |
| Pentose phosphate pathway | Carbohydrate metabolism | Up | 0.094933 | 5.45E-11 | 2.76E-10 |
| Pentose and glucuronate interconversions | Carbohydrate metabolism | Up | 0.104462 | 1.17E-13 | 9.13E-13 |
| Fructose and mannose metabolism | Carbohydrate metabolism | Up | 0.107098 | 9.00E-13 | 5.53E-12 |
| Galactose metabolism | Carbohydrate metabolism | Up | 0.065419 | 2.20E-06 | 6.51E-06 |
| Ascorbate and aldarate metabolism | Carbohydrate metabolism | Up | 0.061718 | 1.10E-05 | 2.86E-05 |
| Starch and sucrose metabolism | Carbohydrate metabolism | Up | 0.07021 | 1.56E-09 | 7.47E-09 |
| Amino sugar and nucleotide sugar metabolism | Carbohydrate metabolism | Up | 0.128733 | 4.82E-23 | 1.99E-21 |
| Pyruvate metabolism | Carbohydrate metabolism | Up | 0.054259 | 2.86E-05 | 6.83E-05 |
| Glyoxylate and dicarboxylate metabolism | Carbohydrate metabolism | n.s. | 0.030094 | 0.050111 | 0.069509 |
| Propanoate metabolism | Carbohydrate metabolism | Up | 0.06232 | 0.000151 | 0.000316 |
| Butanoate metabolism | Carbohydrate metabolism | n.s. | 0.015278 | 0.255587 | 0.301102 |
| Inositol phosphate metabolism | Carbohydrate metabolism | n.s. | -0.00213 | 0.851805 | 0.872086 |
| Oxidative phosphorylation | Energy metabolism | Up | 0.051181 | 0.020645 | 0.030093 |
| Nitrogen metabolism | Energy metabolism | n.s. | -0.01299 | 0.377049 | 0.42112 |
| Sulfur metabolism | Energy metabolism | Up | 0.068606 | 0.001125 | 0.002199 |
| Fatty acid biosynthesis | Lipid metabolism | n.s. | 0.006453 | 0.618706 | 0.656777 |
| Fatty acid elongation | Lipid metabolism | n.s. | 0.028163 | 0.055092 | 0.075205 |
| Fatty acid degradation | Lipid metabolism | n.s. | -0.02263 | 0.084457 | 0.11005 |
| Synthesis and degradation of ketone bodies | Lipid metabolism | Up | 0.035934 | 0.027485 | 0.038749 |
| Steroid biosynthesis | Lipid metabolism | Up | 0.139181 | 7.59E-15 | 6.53E-14 |
| Primary bile acid biosynthesis | Lipid metabolism | Down | -0.03046 | 0.025848 | 0.037049 |
| Steroid hormone biosynthesis | Lipid metabolism | n.s. | 0.015497 | 0.182413 | 0.227355 |
| Glycerolipid metabolism | Lipid metabolism | n.s. | 0.004187 | 0.62623 | 0.656777 |
| Glycerophospholipid metabolism | Lipid metabolism | Down | -0.04357 | 6.60E-09 | 2.70E-08 |
| Ether lipid metabolism | Lipid metabolism | Down | -0.0843 | 2.89E-17 | 3.55E-16 |
| Sphingolipid metabolism | Lipid metabolism | Up | 0.054137 | 2.50E-06 | 7.16E-06 |
| Arachidonic acid metabolism | Lipid metabolism | Down | -0.0828 | 6.31E-12 | 3.39E-11 |
| Linoleic acid metabolism | Lipid metabolism | Down | -0.09231 | 1.27E-12 | 7.28E-12 |
| alpha-Linolenic acid metabolism | Lipid metabolism | Down | -0.0666 | 1.30E-07 | 4.30E-07 |
| Biosynthesis of unsaturated fatty acids | Lipid metabolism | Up | 0.113283 | 3.14E-13 | 2.25E-12 |
| Purine metabolism | Nucleotide metabolism | Up | 0.026387 | 4.35E-05 | 9.83E-05 |
| Pyrimidine metabolism | Nucleotide metabolism | Up | 0.05853 | 4.88E-08 | 1.68E-07 |
| Alanine, aspartate and glutamate metabolism | Amino acid metabolism | Up | 0.082543 | 2.03E-16 | 2.19E-15 |
| Glycine, serine and threonine metabolism | Amino acid metabolism | n.s. | 0.01424 | 0.228055 | 0.272399 |
| Cysteine and methionine metabolism | Amino acid metabolism | Up | 0.094161 | 4.87E-16 | 4.65E-15 |
| Valine, leucine and isoleucine degradation | Amino acid metabolism | Up | 0.041118 | 0.0084 | 0.013134 |
| Valine, leucine and isoleucine biosynthesis | Amino acid metabolism | Up | 0.130233 | 1.78E-08 | 6.95E-08 |
| Lysine degradation | Amino acid metabolism | Up | 0.044241 | 5.96E-05 | 0.000132 |
| Arginine biosynthesis | Amino acid metabolism | n.s. | -0.01563 | 0.218808 | 0.265034 |
| Arginine and proline metabolism | Amino acid metabolism | Up | 0.048054 | 3.21E-05 | 7.46E-05 |
| Histidine metabolism | Amino acid metabolism | n.s. | -0.02253 | 0.091705 | 0.11771 |
| Tyrosine metabolism | Amino acid metabolism | Down | -0.04518 | 0.001982 | 0.003551 |
| Phenylalanine metabolism | Amino acid metabolism | n.s. | -0.0298 | 0.058406 | 0.078483 |
| Tryptophan metabolism | Amino acid metabolism | n.s. | 0.017547 | 0.126106 | 0.159487 |
| Phenylalanine, tyrosine and tryptophan biosynthesis | Amino acid metabolism | Up | 0.096286 | 2.97E-06 | 7.98E-06 |
| beta-Alanine metabolism | Amino acid metabolism | n.s. | -0.00974 | 0.437756 | 0.482654 |
| Taurine and hypotaurine metabolism | Amino acid metabolism | Down | -0.04893 | 0.001471 | 0.002691 |
| Phosphonate and phosphinate metabolism | Amino acid metabolism | Up | 0.117665 | 1.94E-08 | 6.96E-08 |
| Selenocompound metabolism | Amino acid metabolism | Up | 0.034057 | 0.012441 | 0.019105 |
| D-Glutamine and D-glutamate metabolism | Amino acid metabolism | n.s. | 0.014648 | 0.540311 | 0.588187 |
| D-Arginine and D-ornithine metabolism | Amino acid metabolism | n.s. | -0.02078 | 0.573507 | 0.61652 |
| Glutathione metabolism | Amino acid metabolism | Up | 0.065952 | 2.77E-06 | 7.68E-06 |
| N-Glycan biosynthesis | Glycan biosynthesis and metabolism | Up | 0.132212 | 6.96E-23 | 1.99E-21 |
| Various types of N-glycan biosynthesis | Glycan biosynthesis and metabolism | Up | 0.073528 | 4.43E-09 | 1.90E-08 |
| Mucin type O-glycan biosynthesis | Glycan biosynthesis and metabolism | Up | 0.03622 | 0.007841 | 0.012488 |
| Mannose type O-glycan biosynthesis | Glycan biosynthesis and metabolism | n.s. | -0.00572 | 0.682628 | 0.707301 |
| Other types of O-glycan biosynthesis | Glycan biosynthesis and metabolism | Up | 0.038708 | 0.001398 | 0.002615 |
| Glycosaminoglycan biosynthesis - chondroitin sulfate / dermatan sulfate | Glycan biosynthesis and metabolism | n.s. | 0.020912 | 0.299905 | 0.339366 |
| Glycosaminoglycan biosynthesis - heparan sulfate / heparin | Glycan biosynthesis and metabolism | Up | 0.079107 | 1.88E-08 | 6.96E-08 |
| Glycosaminoglycan biosynthesis - keratan sulfate | Glycan biosynthesis and metabolism | Up | 0.085112 | 2.21E-07 | 6.79E-07 |
| Glycosaminoglycan degradation | Glycan biosynthesis and metabolism | n.s. | -0.01835 | 0.270937 | 0.314873 |
| Glycosylphosphatidylinositol (GPI)-anchor biosynthesis | Glycan biosynthesis and metabolism | n.s. | 0.024275 | 0.191678 | 0.23549 |
| Glycosphingolipid biosynthesis - lacto and neolacto series | Glycan biosynthesis and metabolism | n.s. | 0.017156 | 0.297703 | 0.339366 |
| Glycosphingolipid biosynthesis - globo and isoglobo series | Glycan biosynthesis and metabolism | Up | 0.037318 | 0.017943 | 0.026605 |
| Glycosphingolipid biosynthesis - ganglio series | Glycan biosynthesis and metabolism | Up | 0.054642 | 0.001161 | 0.00222 |
| Other glycan degradation | Glycan biosynthesis and metabolism | n.s. | -0.00297 | 0.868341 | 0.878556 |
| Thiamine metabolism | Metabolism of cofactors and vitamins | Down | -0.04094 | 0.005771 | 0.009364 |
| Riboflavin metabolism | Metabolism of cofactors and vitamins | Up | 0.081162 | 0.000146 | 0.000313 |
| Vitamin B6 metabolism | Metabolism of cofactors and vitamins | Up | 0.040639 | 0.017203 | 0.025955 |
| Nicotinate and nicotinamide metabolism | Metabolism of cofactors and vitamins | Down | -0.02871 | 0.002164 | 0.003798 |
| Pantothenate and CoA biosynthesis | Metabolism of cofactors and vitamins | Up | 0.05816 | 1.22E-05 | 3.09E-05 |
| Biotin metabolism | Metabolism of cofactors and vitamins | Up | 0.086557 | 0.004678 | 0.007926 |
| Lipoic acid metabolism | Metabolism of cofactors and vitamins | Down | -0.22808 | 1.07E-17 | 1.53E-16 |
| Folate biosynthesis | Metabolism of cofactors and vitamins | n.s. | 0.022949 | 0.068306 | 0.090374 |
| One carbon pool by folate | Metabolism of cofactors and vitamins | Up | 0.084358 | 3.11E-09 | 1.41E-08 |
| Retinol metabolism | Metabolism of cofactors and vitamins | n.s. | 0.001422 | 0.900754 | 0.900754 |
| Porphyrin and chlorophyll metabolism | Metabolism of cofactors and vitamins | Up | 0.097639 | 8.22E-13 | 5.44E-12 |
| Ubiquinone and other terpenoid-quinone biosynthesis | Metabolism of cofactors and vitamins | Up | 0.101029 | 1.56E-07 | 4.98E-07 |
| Terpenoid backbone biosynthesis | Metabolism of terpenoids and polyketides | Up | 0.164807 | 5.49E-27 | 4.72E-25 |
| Caffeine metabolism | Biosynthesis of other secondary metabolites | Up | 0.072248 | 1.29E-05 | 3.17E-05 |
| Neomycin, kanamycin and gentamicin biosynthesis | Biosynthesis of other secondary metabolites | Up | 0.064093 | 0.000435 | 0.000891 |
| Metabolism of xenobiotics by cytochrome P450 | Xenobiotics biodegradation and metabolism | Down | -0.03407 | 0.0047 | 0.007926 |
| Drug metabolism - cytochrome P450 | Xenobiotics biodegradation and metabolism | Down | -0.0415 | 0.000898 | 0.001797 |
| Drug metabolism - other enzymes | Xenobiotics biodegradation and metabolism | Up | 0.031889 | 0.005008 | 0.008283 |
